# Compartmentalization of Photoreceptor Sensory Cilia

**DOI:** 10.3389/fcell.2021.636737

**Published:** 2021-02-04

**Authors:** Cassandra L. Barnes, Himanshu Malhotra, Peter D. Calvert

**Affiliations:** Department of Ophthalmology and Visual Sciences, Center for Vision Research, SUNY Upstate Medical University, Syracuse, NY, United States

**Keywords:** photoreceptors, transport, rhodopsin, peripheral membrane protein, soluble protein, primary cilia, trafficking, outer segment

## Abstract

Functional compartmentalization of cells is a universal strategy for segregating processes that require specific components, undergo regulation by modulating concentrations of those components, or that would be detrimental to other processes. Primary cilia are hair-like organelles that project from the apical plasma membranes of epithelial cells where they serve as exclusive compartments for sensing physical and chemical signals in the environment. As such, molecules involved in signal transduction are enriched within cilia and regulating their ciliary concentrations allows adaptation to the environmental stimuli. The highly efficient organization of primary cilia has been co-opted by major sensory neurons, olfactory cells and the photoreceptor neurons that underlie vision. The mechanisms underlying compartmentalization of cilia are an area of intense current research. Recent findings have revealed similarities and differences in molecular mechanisms of ciliary protein enrichment and its regulation among primary cilia and sensory cilia. Here we discuss the physiological demands on photoreceptors that have driven their evolution into neurons that rely on a highly specialized cilium for signaling changes in light intensity. We explore what is known and what is not known about how that specialization appears to have driven unique mechanisms for photoreceptor protein and membrane compartmentalization.

## Introduction

**Figure 1:**
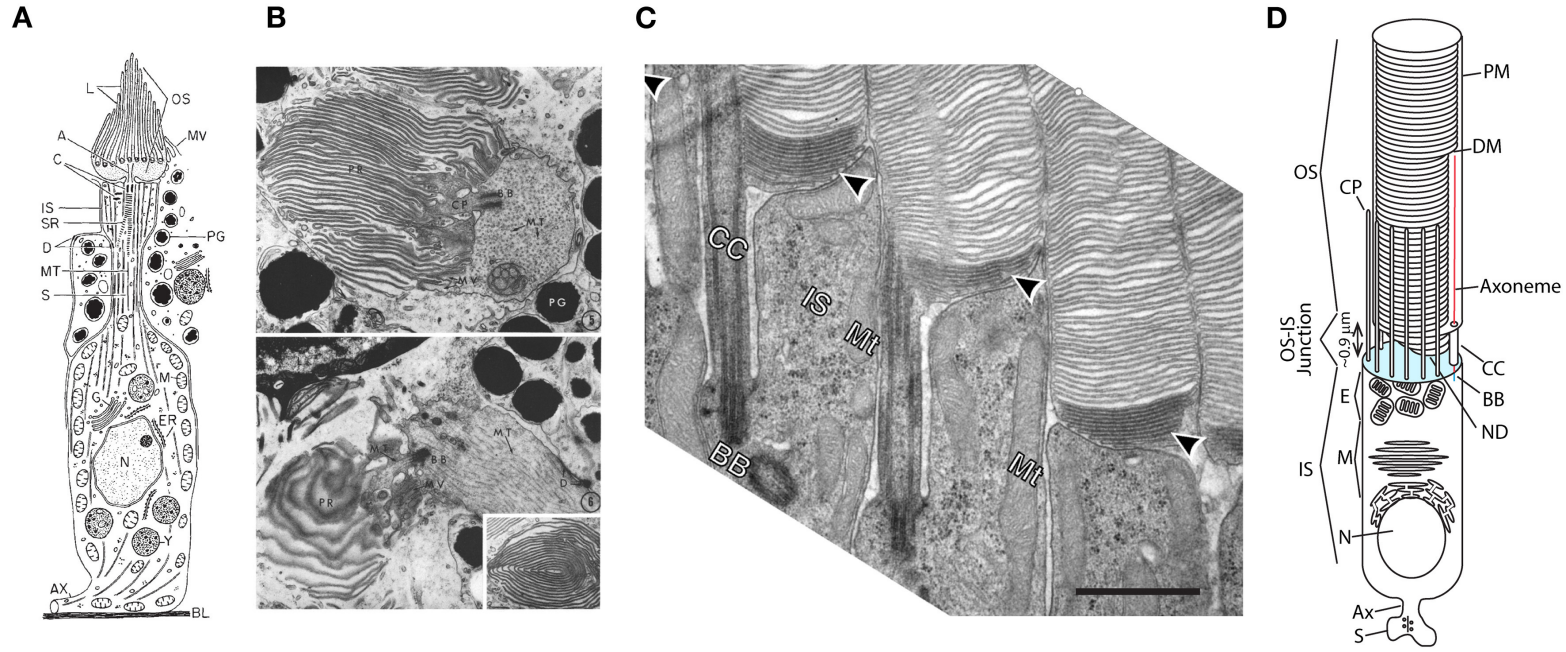
Evolution of ciliary photoreceptors. **(A)** Schematic of an ascidian tadpole cerebral ganglion photoreceptor. Reproduced from Eakin and Kuda (1971). **(B)** Transmission electron micrographs of cerebral ganglion photoreceptors of *Amaroucium constellatum* larva. In the upper panel the section is perpendicular to the membrane lamellae. The lower panel is an image from a section parallel to the lamellar membranes. Reproduced from Barnes (1971). **(C)** Transmission electron micrograph of rod photoreceptors from mouse retina showing the CC, apical membrane region of the IS and nascent and enclosed discs of the OS. Note dense packing of nascent discs (arrowheads) and close juxtaposition of neighboring rods. Scale bar 1 μm. Reproduced from Ding et al. (2015). **(D)** Schematic of an amphibian rod. OS, outer segment; IS, inner segment; S, synaptic spherule; Ax, axon; N, nucleus; M, myoid; E, ellipsoid; CP, calycal process; ND, nascent disc; CC, connecting cilium; DM, mature disc membrane; PM, plasma membrane. Cyan: apical membrane. NDs are open to the extracellular milieu and contiguous with the CC and PM. DMs are enclosed within and separate from the OS PM and each other. Modified from Maza et al. (2019).

Vision in higher vertebrates evolved from ciliary epithelia. Light sensing in animals appears to have evolved from unicellular organisms like the alga *Chlamydomonas* where pigmented eyespots found in the eye organelles reside close to the flagella and support phototaxis by modulating flagellar beating (Gehring, 2004). There are two classes of photoreceptor cells in animals—ciliated and rhabdomeric. Ciliated photoreceptors, where light sensing is located within elaborate organelles having ciliary origins, are found mostly in deuterostomes. Rhabdomeric photoreceptors, where the light capturing organelles are microvilli, are mostly found in protostomes (Gehring, 2004; Lamb et al., 2007). Both ciliary and rhabdomeric photoreceptors originated from epithelial cells that possess cilia and microvilli (Lamb, 2013). The cilium disappeared in rhabdomeric photoreceptors, although the basal bodies were retained, leaving microvilli that are packed with rhabdomeric opsins. Vertebrate photoreceptors possess both ciliary and microvillar structures (Figure 1). The cilia contain rhodopsin and other components of the light transduction machinery localized to expanded ciliary membrane structures (lamellae and discs) in what is known as the outer segments (OS) while the microvilli evolved into support structures, known as calyceal processes, that extend along and in close juxtaposition with the OSs.

Photoreceptors bear a single cilium adapted for the high-fidelity capture and sensation of photons. To meet this demanding function, they have evolved specialized ciliary membrane structures that support efficient photon capture. They support a constant flux of sodium and calcium ions across the ciliary plasma membrane, into the OS. Current flows through the connecting cilium (CC), into the cell body, known as the inner segment (IS), where an outward flux of potassium ions completes the circuit [reviewed in (Pugh and Lamb, 2000)]. The resulting membrane potential changes in a graded manner in response to changes in the light intensity falling on the photoreceptors—leading to graded modulation of the release of neurotransmitter at the synapse. In this way, the epithelial array of photoreceptors produces a receptor potential-encoded readout of the visual scene. A Na^+^, K^+^ antiporter burns ATP to maintain the ionic balance. Photoreceptors and the retina are, thus, amongst the most energetically demanding cells and tissues in the human body (Wong-Riley, 2010; Country, 2017). This metabolic activity leads to high levels of reactive oxygen species and rapid membrane and protein damage. To combat this damage photoreceptors turn over ~10% of their OSs each day (Young, 1967; Young and Droz, 1968; Young and Bok, 1969; Besharse et al., 1977). Therefore, photoreceptors possess one of the most dynamic cilia found in nature. In this review, we examine what is known about the mechanisms by which photoreceptors generate and maintain the OS light signaling compartment and highlight critical knowledge gaps that impede complete understanding of photoreceptor function in health and disease.

## Structure and Function of Ciliated Photoreceptors

A major challenge for photoreceptor light detection is capture and transduction of photons into electrical signals. Unlike chemical signals, which may dwell in the vicinity of sensory cilia for relatively long periods of time, enhancing probability of capture by relevant receptor proteins, photons are elusive, passing through sensory structures at the speed of light. Thus, photoreceptors evolved elaborate membrane systems packed with opsin molecules, the G protein coupled light receptors, to increase the probability of photon capture (Figure 1). Ascidian larva, phylogenetically early chordates, possess photoreceptors where the ciliary membrane evolved into a comb like structure with tens of lamellar membranes (Figures 1A,B) (Barnes, 1971; Eakin and Kuda, 1971).

In higher vertebrates the number of lamellae is expanded to hundreds and tighter organization of the layers of opsin-containing membranes, typical of cone photoreceptors, is imposed (Anderson et al., 1978; Fetter and Corless, 1987). Following cones, rod photoreceptors evolved (Figure 1C), where the lamellae are replaced with thousands of internalized disc membranes that are discontiguous with each other and the OS plasma membrane (Nilsson, 1964; Nir and Pease, 1973; Tsukamoto, 1987; Nickell et al., 2007). Thus, whereas in cones opsins and other membrane proteins may diffuse between lamellae, in rods opsins are confined to the disc membrane into which they are packed for the lifetime of the disc. The lamellae and discs are oriented perpendicular to the path of photons entering the eye, such that photons run a gauntlet of opsin molecules that dramatically increases their probability of capture. The probability of photon capture in photoreceptor cilia is further enhanced by the structure of the photoreceptor ISs through what is known as the Stiles-Crawford effect of the first kind, the namesakes of which first quantified a differential sensitivity to light entering the eye at different angles (Stiles and Crawford, 1933). The photoreceptor ISs act as fiber optic-like waveguides, directing and concentrating light that enters the eye through the pupil into the OS. This waveguiding effect also reduces the capture of scattered photons, thus reducing “glare” and enhancing contrast (Enoch, 1980).

Photoreceptors have, therefore, evolved from ciliated epithelial cells to be super detectors of light with unparalleled efficiency. Indeed, owing to the high density of rhodopsin in each rod OS (Pugh and Lamb, 2000), the remarkable thermal stability of rhodopsin molecules that individually undergo spontaneous isomerization at the remarkably low rate of once in 1,000 years (Ashmore and Falk, 1977; Yau et al., 1979), the high quantum efficiency of rhodopsin [~0.65, (Dartnall, 1972)], and the high gain of phototransduction (Pugh and Lamb, 2000), humans can reliably detect dim flashes that lead to single photon captures in 5 out of 500 rods (Hecht et al., 1942).

## Ciliary Outer Segment Renewal

**Figure 2:**
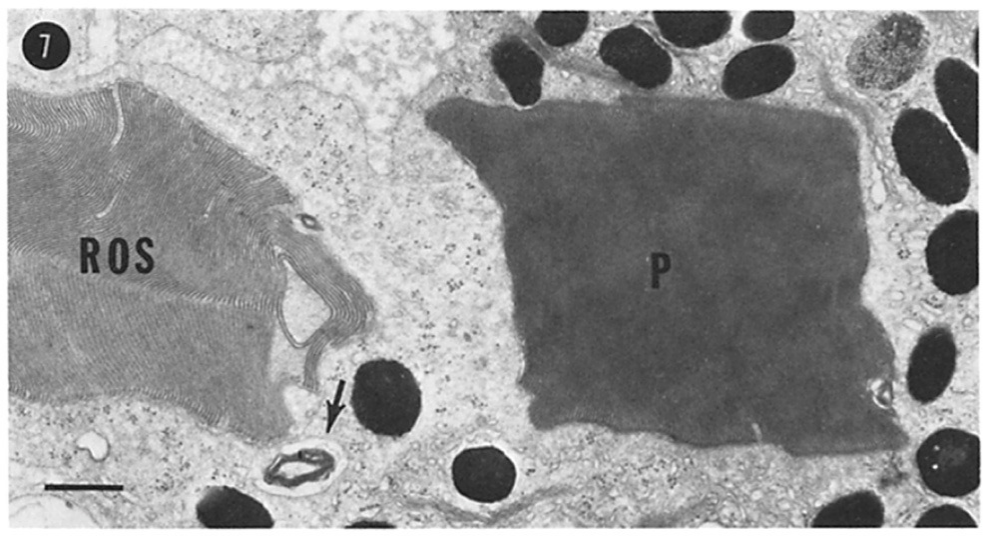
Turnover of photoreceptor OSs. Stack of discs shed from a frog (*Rana pipiens*) rod OS, phagocytosed by a retinal pigment epithelial cell. ROS, rod OS; P, phagosome. Scale bar, 1 μm. Reproduced from Hollyfield et al. (1977).

Unlike primary cilia, where GPCRs and other membrane proteins are delivered and removed from the cilium by secretory and endocytic processes, rhodopsin and other intrinsic membrane proteins are on a one-way ride—once they are packed into disc membranes they never return to the cell body. Instead, in all vertebrate species examined, ~10% of the rod OS length is turned over daily (Young, 1967; Young and Droz, 1968; Young and Bok, 1969; Besharse et al., 1977) through shedding of hundreds of disc membranes at the distal tip of the OS (Figure 2) (Young, 1967; Besharse et al., 1977). Disc shedding is diurnally synchronized, occurring shortly after sunrise in diurnal species, or sunset in nocturnal species. Shed discs are ultimately phagocytosed by the retinal pigment epithelial (RPE) cells lying adjacent to the photoreceptor OSs at the back of the eye (Besharse et al., 1977). Shed discs are replaced by new disc membranes containing rhodopsin and other membrane proteins at the CC/transition zone (Burgoyne et al., 2015; Ding et al., 2015; Volland et al., 2015). Disc morphogenesis (see **Figure 5**) involves a lamellipodium-like, filamentous actin-mediated out pocketing of ciliary membrane (Spencer et al., 2019, 2020; Corral-Serrano et al., 2020) that is initiated by a ciliary ectosome release mechanism (Nager et al., 2017; Phua et al., 2017) whose vesicular scission is suppressed by the tetraspanin protein, peripherin 2 (Salinas et al., 2017).

In lower vertebrates, this daily turnover represents an especially daunting challenge. Amphibian ciliary rod OSs have a diameter of up to 8 μm and length up to 60 μm. The disc repeat frequency is ~30 nm. Approximately 200 discs, or >20,000 μm^2^ of membrane, turn over daily. This turnover includes 300 million rhodopsin molecules as well as ~35 million other molecules essential for light detection. Remarkably, 75% of this turnover occurs within 8 h after the onset of daylight (Besharse et al., 1977). At peak, components of disc membranes and phototransduction machinery are being fed to nascent discs at the base of the OS at the breakneck pace of ~0.6 μm^2^s^−1^. In terms of membrane delivery alone, this rate is equivalent to generating seven primary cilia per minute. Mammalian photoreceptors have a diameter sevenfold smaller than amphibian photoreceptors and are ~50% shorter, thus, the daily demand for membrane and protein delivery to the OS is 100-fold lower.

It is difficult to compare the photoreceptor rate of membrane turnover to the rate of primary ciliary membrane turnover. To our knowledge no direct experiment examining the turnover rate of primary cilium membrane has been published. Indeed, this sort of experiment would be difficult to achieve. Estimates can be made, however. Upon serum starvation, primary cilia of IMCD3 or hTERT-RPE1 cells occurs over the course of ~12 h, over which they extend to stable length of ~5 μm. Thus, the effective membrane delivery rate for photoreceptors is 50-fold (mammalian) to 5,000-fold (amphibian) higher than that required for assembly of a primary cilium in a mammalian epithelial cell.

**Figure 3:**
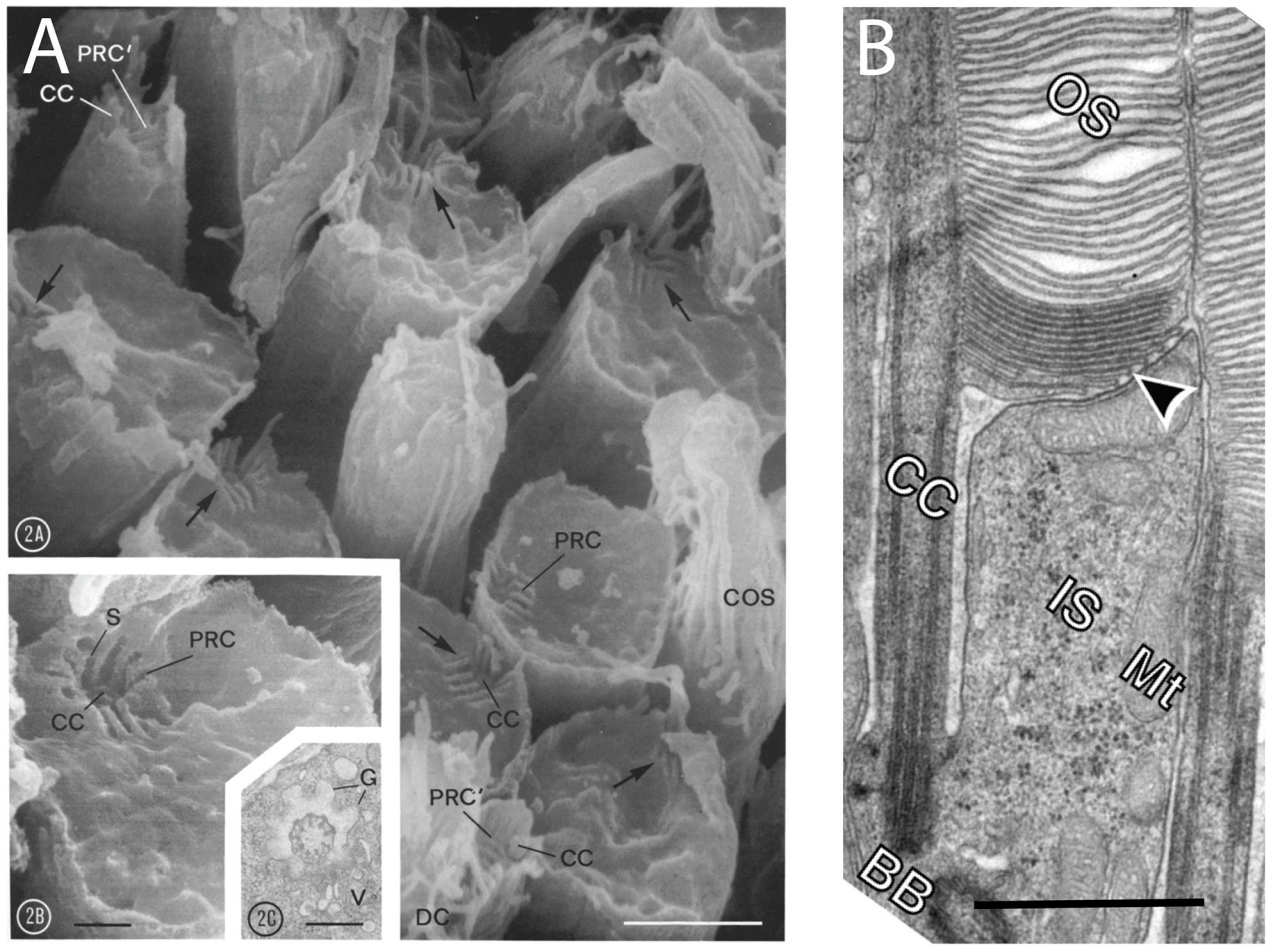
Photoreceptors periciliary membrane systems. **(A)** Scanning electron micrographs of apical surfaces of frog (*Rana pipiens*) rods whose OSs where broken off, exposing the ISs. Note remnants of the CC (CC) recessed in pits at the periphery of the apical membranes (arrows). Membrane infoldings radiating from the connecting cilia are periciliary ridge complexes (PRC). Inset (2C) is a transmission electron micrograph form a section cut perpendicular to the CC. Scale bars, 2A, 2 μm; 2B, 0.5 μm; 2C, 0.5 μm. Reproduced from Peters et al. (1983). **(B)** Transmission electron micrograph through of a mouse rod where the plane of section bisected the CC (CC) and the basal body (BB). A deep ciliary pocket/periciliary membrane extends along one side of the CC (CC) and the nascent disc membranes (arrowhead). The opposite side of the CC abuts the neighboring photoreceptor. IS, IS; Mt, mitochondrion. Scale bare, 1 μm. Reproduced from Ding et al. (2015).

To meet the extraordinary demand for new discs and OS proteins, rod photoreceptors have evolved elaborate periciliary membrane systems that support docking and fusion of rhodopsin transport vesicles (RTCs) (Figure 3). Amphibian photoreceptors possess a periciliary ridge complex consisting of numerous deep folds in the apical membrane surface radiating from the axoneme and basal bodies (Peters et al., 1983) (Figure 3A). The periciliary membrane complex is less elaborate in mammalian photoreceptors, consisting of a single periciliary membrane structure extending along the full length of the CC (Figure 3B). The structure resembles the periciliary pocket associated with primary cilia that develop through the internal, ciliary vesicle mechanism (Benmerah, 2013). Indeed, the photoreceptor cilium develops via this mechanism (Pearring et al., 2013). The pocket associated with mammalian photoreceptors, however, does not completely encircle the CC, presumably due to the placement at the periphery of the cylindrical IS (Figure 3B).

## Structure of the Photoreceptor Connecting Cilium

**Figure 4:**
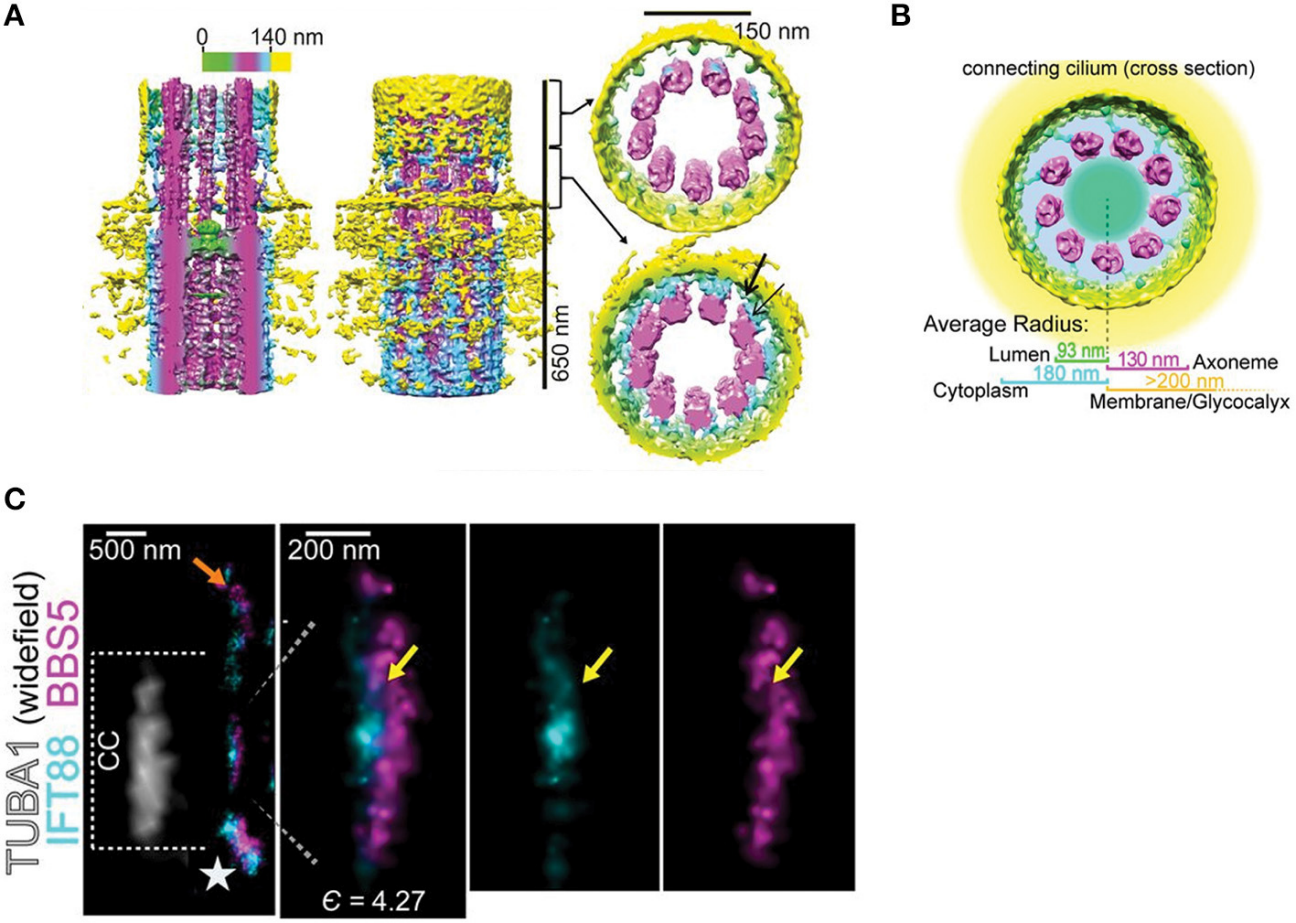
Structure of the photoreceptor CC. **(A)** Surface representations of cryo-EM tomographs subject to 9-fold sub tomograph averaging. Left, section through the ciliary axis, color bar codes distance from center. Middle, entire map. Right upper, distal cross section of the transition zone. Right lower, proximal transition zone cross section shows Y-link-like structures (cyan) projecting between the axoneme (magenta) and the ciliary membrane (yellow). **(B)** Diagram showing the resolution of concentric layers of the CC determined by STORM microscopy, overlaid on cryo-EM tomographic map of ciliary cross section. **(C)** STORM localization maps a CC co-labeled with IFT88 and BBS5. Left panel shows tubulin in bright field to delineate the CC length. Expanded images show close juxtaposition of IFT88 (cyan) and BBS5 (magenta). Right two panels show individual channels. Yellow arrows are suggested to show co-localization. Modified from Robichaux et al. (2019).

The CC consists of basal bodies docked to the apical membrane, a 9+0 microtubule axoneme with and a closely juxtaposed ciliary membrane (Figure 4). Extending between 0.5 μm (amphibians) and 1.5 μm (mammals) from the apical surface, the distal CC gives way to the elaborate OS system of disc (rods) or lamellar (cones) membranes and, in the case of rods, a plasma membrane physically separate from discs. The axoneme extends beyond this point, to between 60 and 100% of the OS length in rods (Brown et al., 1963; Kaplan et al., 1987; Sale et al., 1988; Eckmiller, 1996; Luby-Phelps et al., 2008) and 100% of the cone OS length (Eckmiller, 1996). The photoreceptor axoneme can therefore reach >30 μm in length, although the distal half of the axoneme lose the B subfiber normally found in microtubules (Brown et al., 1963; Steinberg and Wood, 1975; Roof et al., 1991; Insinna et al., 2008).

The CC is thought to correspond to the transition zone (TZ) of primary cilia, although there are some differences. The TZ of all cilia contain a set of evolutionarily conserved proteins, including the Meckel syndrome complex and the nephronophthisis syndrome complex (Williams et al., 2011; Jensen et al., 2015; Li et al., 2016). For a detailed review of TZ proteins and associated diseases, see (Gonçalves and Pelletier, 2017). These proteins are thought to assemble the prototypical Y complexes that bridge the axoneme and ciliary membrane and to form diffusion barriers that regulate protein access to the ciliary compartment (Reiter et al., 2012; Gonçalves and Pelletier, 2017). Mutations in genes encoding these proteins leads to severe syndromic ciliopathies (Reiter and Leroux, 2017). However, mutations in several TZ proteins belonging to the RPGR complex, SPATA7, RPGRIP1, and RPGR, produce photoreceptor specific phenotypes, suggesting they are essential for photoreceptors but not for other cilia and flagella (Roepman et al., 2000; Hong et al., 2001; Murga-Zamalloa et al., 2010). This functional difference led to the hypothesis that the photoreceptor TZ contains a specialization required for assembly of the OS (Dharmat et al., 2018). Two CC regions were proposed, a proximal CC which is homologous to the TZ of primary cilia and the distal CC, which is a photoreceptor-specific extension of the ciliary TZ.

Recent advances in super-resolution fluorescence microscopy and high-resolution cryo-TEM tomography have revealed detailed structure of the TZ and localized protein composition at nanometer resolution (Figure 4) (Gilliam et al., 2012; Yang et al., 2015, 2018; Shi et al., 2017; Robichaux et al., 2019; Sun et al., 2019). An elegant STORM-based study showed detailed localization of 12 distal appendage-associated molecules and 4 additional proteins surrounding the distal appendages (Yang et al., 2018) and super-resolution STED imaging mapped the distribution of TZ complex proteins in primary cilia (Yang et al., 2015). Studies of the CC demonstrated structural details of basal bodies and the axoneme, showing microtubule triplets and doublets, respectively, along with distal appendages (Gilliam et al., 2012; Robichaux et al., 2019). Nine-fold averaging of cryo-EM tomograms revealed Y link-like structures in the lumen of the CC (Figure 4A), a hallmark of the TZ, although the structures appeared to be formed from two independent structures rather than the single structure observed in standard TEM studies in primary cilia (Robichaux et al., 2019). Interestingly, the tomograms also revealed a structure resembling the terminal plate found in motile cilia (Figure 4A). Super-resolution microscopy of immunolabeled proteins revealed concentric layering of the CC (Figure 4B) (Robichaux et al., 2019). Overlapping localization of IFT88, IFT81, and BBS5 distribution across CC was also observed (Figure 4C), suggestive of IFT trains. Interestingly, CEP290, which is present at the base of the primary cilium, was present along the entire CC along with Y-links (Potter et al., 2020). These results have strengthened the notion that the entire length of the CC operates as the photoreceptor transition zone.

A feature common to all cilia is that, despite contiguous plasma and ciliary membranes, the ciliary compartment is physically distinct from the plasma membrane [reviewed in (Garcia et al., 2018)]. This separation is thought to be mediated, in part, by diffusion barriers. For membrane proteins, septin cytoskeletal components may operate as diffusion barriers, retaining ciliary membrane proteins and keeping plasma membrane proteins out. Knockdown of SEPT2 led to normally ciliary confined membrane proteins to exit the primary cilium (Hu et al., 2010). It is possible that two diffusion barriers for membrane proteins exist at the primary cilium base. Super-resolution single molecule tracking revealed that BBSome-mediated transport of agonist activated SSTR3 receptor out of primary cilia required crossing two diffusion barriers, one of which was more stringent (Ye et al., 2018). Super-resolution microscopy studies have shown that the transition zone may be a “waypoint” for proteins where entry or rejection decisions are made (Milenkovic et al., 2015; Shi et al., 2017), perhaps through a matrix/gel-like structure at the distal appendages (Yang et al., 2018). To date the membrane diffusion barrier at the CC has not been identified. Soluble proteins do not appear to be as restricted. Proteins up to ~70 kDa can pass through the ciliary transition zone of primary cilia and the photoreceptor CC without impediment (Calvert et al., 2010; Najafi et al., 2012; Breslow et al., 2013; Lin et al., 2013; Awata et al., 2014).

## Intraflagellar Transport in Photoreceptors

Intraflagellar transport (IFT) is a motor driven process, first observed by Kozminski and Rosenbaum in *Chlamydomonas* sensory flagella (Kozminski et al., 1993; Rosenbaum and Witman, 2002), that is involved in assembly, maintenance, length control, and selective transport of cargo into and out of cilia and flagella (Sedmak and Wolfrum, 2011; Prevo et al., 2017). IFT is mediated by large complexes of proteins that form “IFT trains” which can move in either direction along the cilium. Two classes of IFT have been characterized; IFT-A moves in the retrograde direction, from cilium tip to base, utilizing dynein motors, whereas IFT-B moves in the anterograde direction via kinesin motors (Scholey, 2013; Lechtreck, 2015; Reilly and Benmerah, 2019; Webb et al., 2020). The IFT complex itself is a super-molecular structure that possesses both IFT-A and IFT-B components working together in ciliary protein import and export (Kobayashi et al., 2020). The transport direction and specific cargo depend on switching mechanisms that determine which class of motors engage the axoneme through mechanisms that are not entirely understood (Jordan et al., 2018). Importantly, recent results show that anterograde transport occurs on the B microtubules and retrograde on the A microtubules of the microtubule doublets (Stepanek and Pigino, 2016), thus avoiding traffic jams. IFT malfunction results in syndromic or non-syndromic diseases (Bujakowska et al., 2015), that impact many organ systems, including but not limited to retina, brain, kidney, reproductive organs, and cause obesity [reviewed in Reiter and Leroux (2017)].

In photoreceptors, IFT is involved in the development and maintenance of photoreceptor OSs (Pazour et al., 2002) and is proposed to be involved in the trafficking of opsins and membranes destined for the OS discs or lamellae (Pearring et al., 2013; Wheway et al., 2014; Imanishi, 2019). IFT52, 57, 88, and 20 have been identified along the OS axoneme and within the IS of *Xenopus* photoreceptors (Luby-Phelps et al., 2008) and IFT88 and 81 in the CC of mouse photoreceptors (Robichaux et al., 2019) (Figure 4). Mutations in IFT proteins have been shown to cause mislocalization of rhodopsin and accumulation of membrane vesicles in the IS. For example, IFT20 is implicated in trafficking of rhodopsin from the Golgi to the base of the OS (Follit et al., 2006) and mutation in IFT122 in zebrafish appeared to delay opsin transport to the OS, causing progressive photoreceptor degeneration (Boubakri et al., 2016). IFT proteins have also been found to be associated with basal bodies prior to elaboration of the OS in developing retinal photoreceptors, suggesting IFT proteins may be playing a role in early photoreceptor ciliogenesis (Sedmak and Wolfrum, 2011). While many of these studies show mislocalization of rhodopsin and conclude that the mutations impact its trafficking, it is not clear that they represent direct, causal relationships. In addition to uncoupling of cargo from IFT, mistrafficking could result from improper ciliogenesis or perturbation in the homeostasis of the CC. Moreover, to date there is no direct evidence that opsins transport via IFT within the CC.

The dynamics of IFT proteins and co-transport of IFT cargos within cilia and flagella have been exhaustively explored in various model systems including mammalian cells, *Chlamydomonas* flagella and sensory neurons of *C. elegans*. These studies have led to detailed, quantitative descriptions of the frequency, kinetics and size of IFT trains and their cargoes (Pigino et al., 2009; Taschner et al., 2014, 2018; Stepanek and Pigino, 2016; Vannuccini et al., 2016; Jordan et al., 2018; Yang et al., 2019; Kiesel et al., 2020). In *Chlamydomonas*, it is estimated that, on average, ~10–12 IFT trains are present per flagellum (Vannuccini et al., 2016; Wingfield et al., 2017). There appear to be two sizes of trains, short trains averaging ~250 nm and long trains of ~650 nm (Pigino et al., 2009; Vannuccini et al., 2016). The rate off IFT transport in *Chlamydomonas* flagella on average is ~2 μm s^−1^ anterograde and ~3 μm s^−1^ retrograde (Wingfield et al., 2017). IFT rates in primary cilia have been estimated to be ~0.6 μm s^−1^ anterograde and ~0.3 μm s^−1^ retrograde (Follit et al., 2006; Ye et al., 2013; Broekhuis et al., 2014; Lee et al., 2018a). Such quantification allows estimates of the cargo carrying capacity and rate of cargo delivery by IFT. No direct high-resolution studies of IFT dynamics within photoreceptor connecting cilia have been achieved, however, representing a substantial knowledge gap.

There are several studies suggesting non-IFT trafficking of cargo may play a larger role than previously appreciated. In *Chlamydomonas* flagella IFT mediated trafficking of tubulin is predominant when flagella are extending, but diffusion of tubulin within the flagellum predominates during steady-state axonemal turnover (Craft et al., 2015). Soluble EGFP or PAGFP and concatemers of up to three of these proteins diffuse into *Xenopus* photoreceptor OS, showing that soluble proteins as large as 80 kDa do not require active IFT transport (Calvert et al., 2010; Najafi et al., 2012). Similar results were found for soluble proteins in primary cilia (Breslow et al., 2013). Diffusion was also reported to be a major mode of transport for intrinsic membrane proteins in cilia (Ye et al., 2013; Milenkovic et al., 2015; Lee et al., 2018a).

## The BBSome in Photoreceptors

Bardet-Biedl syndrome (BBS) is a ciliopathy resulting from mutations in multiple genes that lead to retinal degeneration, polydactyly, morbid obesity and kidney disfunction among other disorders (Hernandez-Hernandez and Jenkins, 2015; Nachury, 2018; Wingfield et al., 2018). Eight of these genes encode proteins that form the BBSome, a complex involved in protein trafficking and ciliogenesis, including photoreceptor morphogenesis (Hsu et al., 2017). The BBSome shares common structural elements with COPI, COPII, and clathrin coats, indicating a role in membrane and membrane protein transport. The BBSome has been shown to be involved in entry of intrinsic membrane proteins into cilia. For example, the GPCRs SSTR3 and MCHR1 failed to localize to primary cilia of hippocampal neurons from BBS2^−/−^ and BBS4^−/−^ mice (Berbari et al., 2008).

Recent studies suggest the BBSome plays a major role in removal of membrane and soluble proteins from cilia. Primary cilia from central neurons in mice where BBS2, BBS4, or BBS7 are knocked out resulted in accumulation of the dopamine 1 receptor (DP1) in cilia and agonist-induced transport of DP1 out of the cilia was reduced (Domire et al., 2011; Zhang et al., 2013). Primary cilia in mouse embryonic fibroblasts (MEFs) from BBS7^−/−^ and BBS3^−/−^ mice accumulate Patch1 and Smo receptors in the absence of smoothened agonist, SAG (Zhang et al., 2011b, 2012). Smo accumulates in cilia of BBS1^−/−^ RPE1 cells in the absence of SAG ligand while GPR161 was retained in cilia after ligand treatment (Nozaki et al., 2018). These results show that BBSome transport is required for removal of these receptors from primary cilia. BBSome-dependent removal of activated SSTR3 and Smo from primary cilium of IMCD3 cells requires the adapter proteins β-arrestin2, which binds to activated, phosphorylated GPCRs, and Tulp3 (tubby-like protein 3), which is a PI(4,5)P2 binding protein (Ye et al., 2018). In addition, ubiquitination of GPCRs has been shown to precede their β-arrestin2-BBSome mediated removal from cilia (Desai et al., 2020; Shinde et al., 2020).

In addition to intrinsic membrane proteins, BBSome may regulate the removal of peripheral membrane and soluble proteins from cilia. In WT *Chlamydomonas*, the peripheral membrane protein Phospholipase D (PLD) is excluded from flagella. In *BBS1,4, and 7* mutant *Chlamydomonas* PLD slowly accumulates in flagella (Lechtreck et al., 2013; Liu and Lechtreck, 2018). The soluble protein Carbonic anhydrase 6 (CAH6) is excluded from one of the two *Chlamydomonas* flagella. However, *BBS1* mutant *Chlamydomonas* loses this asymmetry and CAH6 was found to be present in both flagella, suggesting the BBSome removes CAH6 selectively from one flagellum (Yu et al., 2020). Overall, these studies provide strong evidence that the BBSome complex is involved in removal of proteins from cilium and that the BBSome is a highly selective regulator of ciliary protein complement.

Retinal degeneration is the most common phenotype in BBS; almost all patients develop retinitis pigmentosa. BBS has, therefore, been intensely studied in the context of retinal degeneration. Animal and cell models, spanning unicellular organisms to non-human primates, have been developed to decipher the role of BBSome proteins in photoreceptor biology (Wingfield et al., 2018; Peterson et al., 2019). Knockout of BBSome proteins in mice recapitulates progressive degeneration of photoreceptors observed in human patients. Onset of degeneration varied among studies with mutations or knockouts of different BBSome proteins (Nishimura et al., 2004; Abd-El-Barr et al., 2007; Davis et al., 2007; Swiderski et al., 2007; Simons et al., 2011). In parallel to studies in primary cilia, recent studies have emphasized the role of the BBSome in removal of non-ciliary proteins from the OS. Quantitative proteomic analysis of photoreceptor OS of WT and BBS17 mutant mice showed enrichment of 139 proteins in the OS of the mutant, including a 3-fold increase in Stx3 and Munc18-1/Stxbp, IS proteins involved in RTC fusion with periciliary membranes (Datta et al., 2015). Only eight proteins normally localized to the OS showed reduced OS localization. OS proteins, including Rhodopsin, peripherin2, PDE6α,β, and Rom-1, remained unchanged. Accumulation of Stx3 in the OS was later confirmed in BBS8^−/−^, BBS4^−/−^, and BBS1^−/−^ mice as well (Hsu et al., 2017; Dilan et al., 2018). These results show that the BBSome, like in primary cilia and flagella, is a regulator of OS protein complement.

An important difference between photoreceptors and primary cilia or flagella, however, is the role of the BBSome in regulating the trafficking of GPCRs into and out of ciliary compartment. Rhodopsin content within the rod OS does not appear to be strongly regulated by the BBSome. While knockout of several BBSome proteins results in mislocalization of rhodopsin, it is important to note that this mislocalization is either incomplete, where the majority of rhodopsin properly localizes to the OS (Abd-El-Barr et al., 2007; Pretorius et al., 2010; Jiang et al., 2016), or is accompanied by major structural disruption, or complete absence of the OS (Nishimura et al., 2004; Abd-El-Barr et al., 2007; Simons et al., 2011). In the case where there is loss of the OS structure, it is impossible to ascertain if the mislocalization is due to rhodopsin transport deficits rather than from lack of the ciliary destination. In the case where there is slight mislocalization with the majority of rhodopsin properly localized to the OS, it is hard to make a case that the BBSome is playing a major role. There is good evidence that rhodopsin localizes to photoreceptor cilia via a mechanism distinct from that of other GPCR localization to primary cilia (Geneva et al., 2017). The c-terminal VxPx motif that is required for OS targeting of rhodopsin inhibits GPCR targeting to primary cilia (Geneva et al., 2017). This is not surprising since the VxPx motif is not recognized by the BBSome (Klink et al., 2017). Replacing the IC3 loop of rhodopsin with that of SSTR3 and removal of the c-terminal VxPx motif resulted in enhanced ciliary localization of the chimeric rhodopsin heterologously expressed in epithelial cells. A recent study has shown that the CTS within the IC3 loop of SSTR3 provides strong binding to a recombinant BBSome core complex, but that the IC3 loop of 5-HT-6 receptor, which is similar to that of rhodopsin, provided only weak interaction (Klink et al., 2017). This weak interaction, as well as the presence of an FR motif in rhodopsin helix 8, which also has weak affinity to the BBSome (Klink et al., 2017; Yang et al., 2020), may support the low presence of rhodopsin in primary cilia (Trivedi and Williams, 2010; Trivedi et al., 2012; Wang et al., 2012; Geneva et al., 2017; Chadha et al., 2019).

In conclusion, the role, if any, of BBSome in rhodopsin OS localization or removal is not clear. However, there is evidence that it plays a role in cone opsin transport to the COS (Abd-El-Barr et al., 2007; Bales et al., 2020).

## Intrinsic Membrane Protein Compartmentalization Within the Ciliary ROD Outer Segment

**Figure 5:**
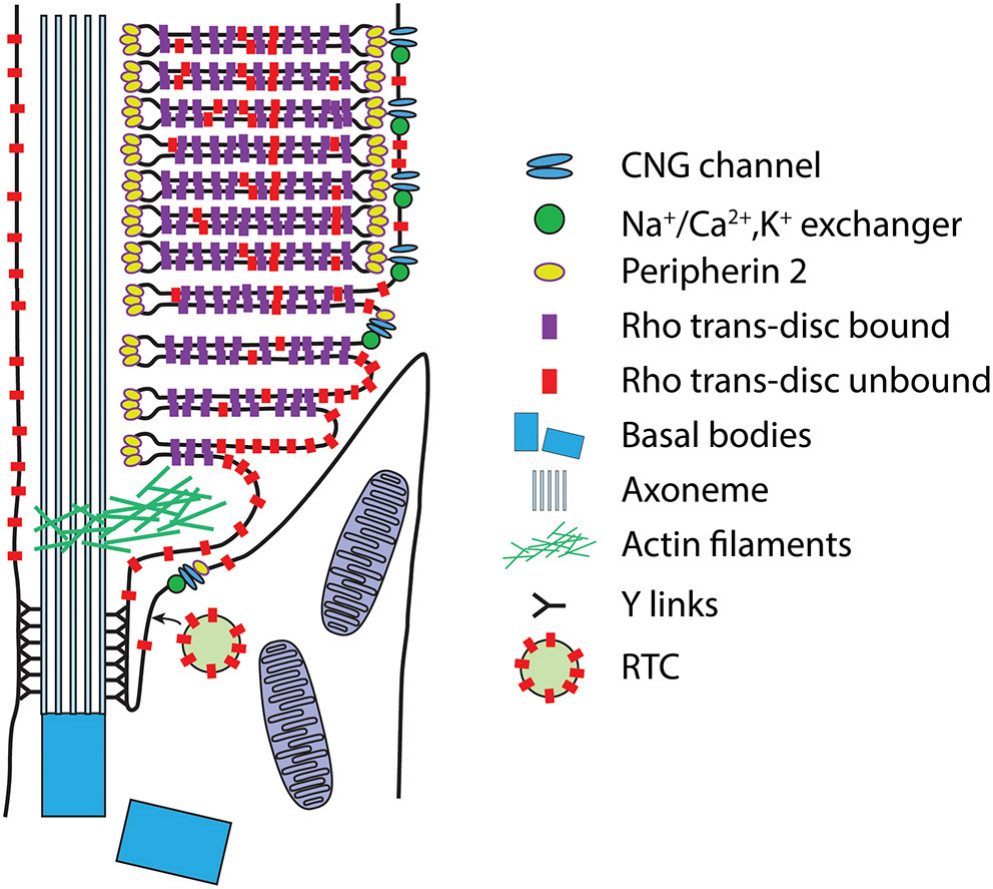
Intrinsic membrane protein compartmentalization within the OS. Schematic of a mammalian rod in the region of the CC. Intrinsic membrane proteins are thought to be delivered to the apical/periciliary membrane on rhodopsin transport carrier (RTC) vesicles, where they fuse (see text for details). Membrane proteins then enter the CC/transition zone, possibly mediated by the BBSome. Proteins destined for disc and plasma membrane transport to the lamellar membranes most likely by diffusion. Eventually the nascent disc membranes are enclosed by the plasma membrane through a mechanism that separates plasma membrane proteins, the CNG gated channel-Na^+^/Ca^2+^,K^+^ exchanger complex, from the disc proteins, rhodopsin and peripherin 2, which likely occurs during peripherin 2-Rom1-mediated disc rim expansion (see text). Note that this schematic is not meant to be an exhaustive representation of all OS membrane protein transport, or of disc morphogenesis.

Compartmentalization of intrinsic membrane proteins within the photoreceptor OS is an extraordinarily demanding problem (Figure 5). It has been proposed that the OS serves as a default destination for membrane proteins; those that are localized elsewhere containing targeting information within their sequence and structure (Baker et al., 2008). Considering the sheer mass of proteins that must travel to the OS, this would seem to be an efficient and perhaps necessary mechanism. However, some membrane proteins contain specific ciliary localization sequences that are required for their transport to the OS (Mazelova et al., 2009; Salinas et al., 2013), suggesting the default pathway is not the only pathway.

There are two distinct secretory pathways involved in membrane protein trafficking, the conventional and unconventional pathways. The conventional pathway refers to proteins that are transported from the ER to the Golgi and bud off the Golgi into transport vesicles. Proteins going through the unconventional pathway exit the ER and bypass the Golgi. A convenient way to differentiate between these pathways is to examine sugars that are post translationally added to some proteins. Glycosylated proteins that take the conventional route acquire endoglycosidase H (Endo H) resistance as they pass through the medial trans-Golgi (Rabouille et al., 1995). The conventional pathway accounts for the majority of proteins traveling to OSs including Rhodopsin (Nathans, 1992; Nickell et al., 2007), Rom1, the CNGα1/CNGβ1 complex, PRCD, and GC1. Both PRCD and GC1 require rhodopsin for their stability and trafficking to the OS (Pearring et al., 2015; Spencer et al., 2016). A recent study confirmed trafficking of GC1 through the conventional pathway (Pearring et al., 2020).

### Rhodopsin Transport and the Conventional Pathway to the Cilium Base

Rhodopsin's VxPx motif is considered to be its primary localization signal and is required for binding to the small GTPase Arf4 (Deretic et al., 2005; Mazelova et al., 2009). Interestingly, VxPx may not be essential for OS localization of rhodopsin. A transgenic study expressing human rhodopsin in *Xenopus* found that there is a secondary signal in rhodopsin within amino acids 322–336 that is responsible for its mislocalization when the VxPx motif is absent (Lodowski et al., 2013). When both the primary and secondary signals are removed, rhodopsin again localizes to the OS, providing supporting evidence for the default OS trafficking pathway.

Rhodopsin trafficking from the Golgi to the periciliary membrane has been thoroughly studied and reviewed (Deretic and Wang, 2012; Wang and Deretic, 2014; Deretic et al., 2019) and will be covered briefly here. The Arf family of small GTPases are involved in various cellular processes including membrane trafficking (Donaldson, 2005). When activated by the GEF GBF1, Arf4 can bind to the VxPx of rhodopsin (Wang et al., 2017). The Arf GAP ASAP1 then binds to rhodopsin-Arf4 and recruits Rab11a and FIP3 which aid in the GTP hydrolysis of Arf4 inactivating and dissociating Arf4 from the rhodopsin-ASAP1-Rab11a-FIP3 complex. Rabin8 is recruited to the developing vesicle and leads to the formation of the Rab11-Rabin8-Rab8 module. The Rab11-Rabin8-Rab8 module recruits the SNARE component VAMP7 into the budding secretory vesicles, called rhodopsin transport carriers (RTCs) (Deretic et al., 2004; Wang and Deretic, 2014), making them competent for fusion with the plasma membrane near the base of the cilium containing the partner SNARE components syntaxin 3 and SNAP25 (Kandachar et al., 2018).

The role of the Arf4 pathway in rhodopsin transport is controversial. While the Arf4-based rhodopsin trafficking pathway has been extensively characterized *in vitro* and in amphibians (Deretic et al., 1998, 2005; Moritz et al., 2001; Mazelova et al., 2009; Wang et al., 2012, 2017; Wang and Deretic, 2015), recent studies have called the requirement of Arf4 into question. Conditional knockout of Arf4 in mouse photoreceptors had no effect on rhodopsin localization (Pearring et al., 2017). Additionally, removing Rab8a and Rab11a from the mouse retina individually or concurrently had no effect on OS protein localization or the ability of the mutant mice to respond to light as measured by ERG (Ying et al., 2016). In light of these results, it has been proposed that, in the mouse where the membrane trafficking requirements are lower, another Arf may be able to partially compensate (Wang et al., 2017). Alternatively, it has been speculated that another Arf pathway, perhaps utilizing Arf5, that is independent of Rab8a and Rab11a is operating in mouse (Ying et al., 2016). Further studies into rhodopsin trafficking pathways in mouse models will be necessary to resolve these discrepancies.

Rhodopsin's transport in RTCs from the Golgi to the base of the cilium is thought to require IFT20 which moves along microtubules (Keady et al., 2011), likely via KIFC1 (Kinesin family member C1) which has been shown to interact with ASAP1, or possibly dynein motors (Krock et al., 2009). Indeed, in cultured primary ciliary cells knockdown of *Kifc1* resulted in a lack of cilia formation and accumulation of ASAP1 and receptors Smo and SSTR3 at the Golgi (Lee et al., 2018b). IFT20 is somewhat different from other components of the canonical IFT-B anterograde complex as it is not solely localized to the base and length of the cilium, it is also found at the Golgi. IFT20 is likely an adaptor that binds rhodopsin and serves to transport the RTCs from the TGN to the base of the periciliary membrane (Keady et al., 2011). Cultured cells where *Ift20* is knocked out fail to develop cilia (Follit et al., 2006; Takei et al., 2018), suggesting that IFT20's main role is likely in regulating ciliogenesis, maintenance of the cilium, and trafficking of ciliary components to the basal body.

### Rhodopsin Transport Across the Cilium Base and Within the Connecting Cilium

Rhodopsin's mode of transport across the CC has yet to be determined. There are two main camps: The first is that RTCs themselves are transported through the CC via motor driven transport and fuse with nascent discs within the enclosed OS plasma membrane (Chuang et al., 2007; Gilliam et al., 2012). However, RTCs average 250–300 nm (Deretic and Papermaster, 1991; Deretic and Mazelova, 2009), making them far too large to pass through any part of the CC. Moreover, recent careful EM studies have clearly shown nascent discs to be open to the extracellular milieux (Burgoyne et al., 2015; Ding et al., 2015; Volland et al., 2015). Finally, immunogold labeling experiments have shown rhodopsin to be located in the ciliary membrane of mouse photoreceptors with little in the lumen of the cilium (Wolfrum and Schmitt, 2000; Burgoyne et al., 2015; Chadha et al., 2019).

The second camp posits that RTCs fuse with membranes at the periciliary ridge complex (Papermaster et al., 1986). It is then thought that rhodopsin is transported by IFT along the plasma membrane of the CC toward the site of disc formation (Krock and Perkins, 2008; Bhowmick et al., 2009). However, the sheer mass of rhodopsin that must be transported does not appear to support the IFT hypothesis. Ten percent of the OS of each rod is renewed daily (Besharse, 1986). Based on the total OS rhodopsin content of 3 × 10^9^ molecules (amphibian) and 1 × 10^8^ molecules (mammalian) (Pugh and Lamb, 2000), the rate of rhodopsin transport through the CC is on the order of 3500 molecules s^−1^ in the frog, and 100 molecules s^−1^ in mouse. Notably, it has been shown that disc morphogenesis and rhodopsin transport is not constant, but rather undergoes a burst of activity where 70% of new discs are formed within the first 8 h of daylight in frog (Besharse et al., 1977) and a smaller variation in delivery upon onset of darkness in mouse (Volland et al., 2015). Thus, within this time-period the rhodopsin delivery rate is closer to 7,300 molecules s^−1^ in amphibians and 100–200 molecules s^−1^ in mouse. This would require high frequency IFT transport within the CC, and significant recycling of IFT complexes, for which there is no evidence.

Aside from the above quantitative argument, evidence for active transport of rhodopsin has been variable. *KIF3A* conditionally knocked out in mouse photoreceptors resulted in the mislocalization of opsin, leading to the conclusion that KIF3A, and by extension IFT, is essential for rhodopsin transport within the CC (Marszalek et al., 2000; Jimeno et al., 2006). In contrast, in another rod specific *KIF3A* knockout mouse rhodopsin and many of the other phototransduction proteins appeared to transport normally to the OS for 2–4 weeks, after which rods degenerated (Avasthi et al., 2009). Similarly, retina specific tamoxifen inducible deletion of KIF3A and IFT88 in adult mice showed normal localization of rhodopsin for 2 weeks before degeneration of photoreceptors while the same deletion from embryonic stage resulted in lack of assembly of the CC (Jiang et al., 2015b). Therefore, in rods, IFT does not appear to be essential for rhodopsin transport within the CC and opsin mislocalization is likely an indirect effect of the failure to properly form and maintain a cilium. Importantly knockout of *KIF17*, the only other known anterograde IFT motor, in conjunction with knockout of *KIF3A* in rods does not prevent rhodopsin trafficking to the ROS, thus showing that there is no compensatory expression of motors (Jiang et al., 2015a).

Interestingly, there was major mislocalization of phototransduction components in cone specific *KIF3A* knockout mice (Avasthi et al., 2009), and KIF3B dominant negative mutant causes accumulation of large vesicles in cone IS and disruption of cone OS morphogenesis (Insinna et al., 2009). These results suggest that IFT is required for transport of opsin and other phototransduction proteins in cones, but not in rods.

The majority of evidence, thus, seems to point to an IFT-independent rhodopsin transport mechanism in rods. We propose that diffusion along the ciliary membrane is the primary mode of rhodopsin transport within the CC. In support of this idea our lab has shown that rhodopsin transport is exclusively by diffusion when heterologously expressed in the primary cilia of IMCD3 cells (Lee et al., 2018a). Indeed, SSTR3 and Smo receptors have also been shown to move mostly by diffusion in IMCD3 cilia (Ye et al., 2013; Lee et al., 2018a). With an average diffusion coefficient of 0.23 μm^2^s^−1^ along the ciliary membrane (Lee et al., 2018a), a rhodopsin molecule could traverse a 1 μm long CC in ~2 s, easily rapid enough to account for rhodopsin delivery to nascent discs. Together, these studies have thrown the notion that GPCRs transport exclusively by IFT within cilia into question.

**Figure 6:**
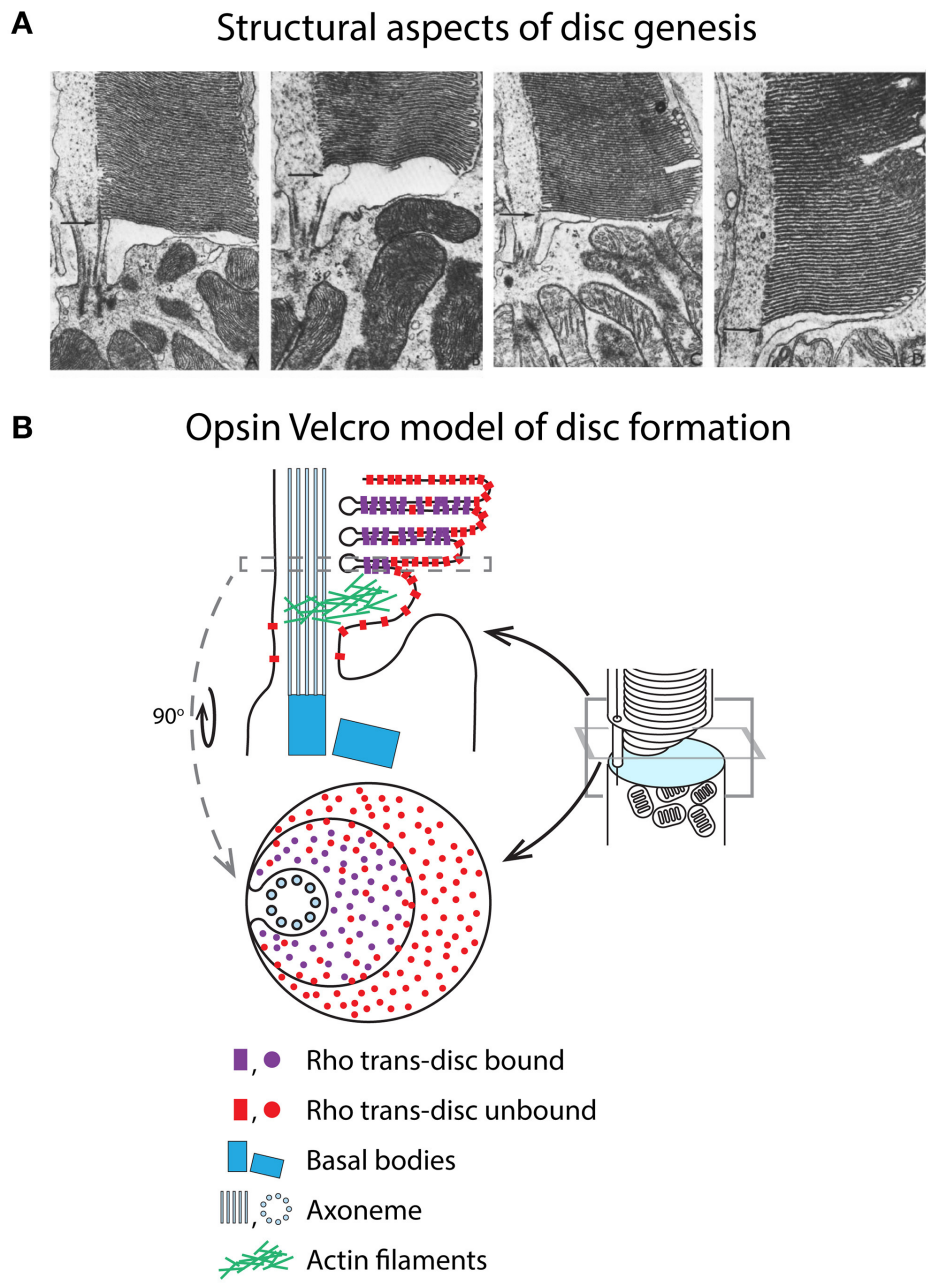
The opsin Velcro model of rhodopsin enrichment in disc membranes. **(A)** Series of transmission EM images showing how nascent disc morphogenesis is thought to proceed. Note the close juxtaposition (~2–3 nm) of extracellular membrane leaflets between the previous and new lamellae as the membrane of the new lamella elongates. Reproduced from Steinberg et al. (1980). **(B)** Rhodopsin density in the disc membranes is twice that in the plasma membrane, indicating that it is not efficiently separated into disc membranes. One possible explanation for this asymmetry is that rhodopsin self-associates at is extracellular N-termini. This may result in a Velcro-like coupling of the nascent disc membranes and producing a self-binding sink driving disc enrichment. Affinity would not be expected to be high for this interaction since it only produces a twofold difference in disc vs. plasma membrane density. This interaction may also help drive disc morphogenesis. For simplicity, rhodopsin cis dimers (in the same membrane) are not depicted in this schematic.

It is well-documented that the density of rhodopsin is ~two-fold higher in the disc membranes than in the OS plasma membrane (Molday and Molday, 1987), which is contiguous with the CC membrane. This raises the question of what is the driving force behind concentrating rhodopsin in the discs if IFT is not involved? One enticing possibility is that rhodopsin is drawn to the nascent discs by a binding sink created during disc elaboration. Rhodopsins are thought to form dimers between adjacent nascent disc membranes (Fliesler et al., 1985; Hubbell et al., 2003; Murray et al., 2009), interacting at their extracellular faces, which may enhance close juxtaposition of extracellular membranes by “Velcroing” them together (Figure 6). The propensity for rhodopsins to form cis dimers (Ploier et al., 2016; Zhang et al., 2016b), within the same disc membrane, may further drive concentration by recruiting rhodopsins into higher order oligomers.

### Peripherin 2 and the Unconventional Pathway

Proteins transported via the unconventional secretory pathway exit the ER-Golgi prior to glycosylation modifications that take place in the medial to trans-Golgi apparatus (Tian et al., 2014). Peripherin 2, a tetraspanin protein that is localized to the rims of discs (Figure 5) and is required for proper OS formation, appears to be transported through the unconventional pathway. Ciliary targeting of peripherin 2 is dependent on COPII-mediated exit from the ER (Tian et al., 2014) and appears to require a signal in the C-terminus that includes Valine 332 (Tam et al., 2004; Salinas et al., 2013, 2017; Molday and Goldberg, 2017; Conley et al., 2019a). Peripherin 2 transport also appears to require interactions with SNARE machinery (Zulliger et al., 2015) and may be trafficked to the OS through a mechanism involving the late endosome (Otsu et al., 2019). However, peripherin 2 appears to take the conventional route ~30% of the time in mice, likely via hetero-oligomerization with rom-1 (Conley et al., 2019b). Until recently, peripherin 2 was the only OS-destined protein identified that is transported through the unconventional pathway. However, evidence suggests that R9AP traffics to the OS independently of rhodopsin, suggesting that it may transport via the unconventional secretory pathway (Pearring et al., 2014) and a recent study has shown that ABCA4 also traffics via the unconventional pathway (Pearring et al., 2020).

## Roles of Phosphoinositides in Protein Enrichment in the Photoreceptor Cilium

Phosphoinositide phospholipids are known to be important for various cellular processes, creating specialized membrane compartments, recruiting proteins with phosphoinositide binding capacities, trafficking of membrane proteins, and serving as precursors for cellular second messengers. For a comprehensive review of phosphoinositides in the context of the retina, see (Wensel, 2020). Here we will focus on PI4P and PI(4,5)P_2_, the two most abundant phosphoinositides in photoreceptors (Finkelstein et al., 2020).

PI4P is enriched in the Golgi via dephosphorylation of PI(4,5)P_2_ and PI(3,4)P_2_ or phosphorylation of PtdIns (De Matteis et al., 2002; Liu and Bankaitis, 2010) and is thought to be important in trafficking through and vesicle budding from the Golgi and vesicular trafficking to the plasma membrane (Godi et al., 2004; Lenoir and Overduin, 2013). While PI4P is the predominant phosphoinositide present in the Golgi, there is also a small pool of PI(4,5)P_2_.

Ezrin and moesin interact with transmembrane proteins, PDZ-containing proteins, the cytoskeleton, and bind membranes via PI(4,5)P_2_ (Bretscher et al., 2002). These shared functions indicate they may be important in the trafficking of membrane proteins. In support of this notion, both ezrin and moesin were shown to be present on RTCs, particularly at the site of vesicle docking near the IS/OS junction (Deretic et al., 2004). Altering biosynthesis of acidic phospholipids ultimately resulting in the hydrolysis of PI(4,5)P_2_ led to a reduced association of ezrin and moesin with RTCs and interfered with RTC docking and fusion at the base of the CC (Deretic et al., 2004).

A mouse at Jackson Laboratories named tubby had a spontaneous mutation and an obesity phenotype (Coleman and Eicher, 1990). The tubby gene product is expressed in the brain and retina, and *tubby* mice have both retinal and cochlear degeneration (Ohlemiller et al., 1995) and reduced fertility (Ohlemiller et al., 1998). At least four other tubby-like proteins (TULPs) have since been identified that contain a conserved C-terminal tubby domain which can interact with PtdIns(3,4)P_2_, PtdIns(4,5)P_2_, and PtdIns(3,4,5)P_3_ (Santagata et al., 2001). Tubby proteins interact with the plasma membrane by binding PI(4,5)P_2_ and are released when PI(4,5)P_2_ is hydrolyzed (Santagata et al., 2001). It has been demonstrated that activation of Gα_q_ causes tubby to leave the plasma membrane via a PLC-β mediated mechanism of PI(4,5)P_2_ hydrolysis (Santagata et al., 2001).

Tulp1 is exclusively found in photoreceptors, thus it is no surprise that *tulp1* mice have retinal degeneration but lack the cochlear defects and the obesity phenotype seen in tubby mice (Ikeda et al., 2000). Mutations in TULP1 contribute to ~5% of total RP cases (Gu et al., 1998) and *tulp1* mice accumulate vesicles in the interphotoreceptor matrix (Hagstrom et al., 1999) similar to those seen in the *rds* and *pcd* mouse. TULP1 interacts with F-actin (Xi et al., 2005) and dynamin (Xi et al., 2007) in photoreceptors, suggesting that it may play a role in vesicular trafficking to the periciliary plasma membrane. Similar functions have been identified for TULP3 in primary cilia where RNAi knockdown of TULP3 decreased trafficking of some GPCRs to cilia without affecting ciliogenesis. The interaction of TULP3 with IFT-A and phosphoinositides is required for trafficking of particular GPCRs to cilia (Badgandi et al., 2017), and it has been shown that Tub and TULP2 can also bind IFT (Mukhopadhyay et al., 2010). Knockdown of the inositol polyphosphate 5' phosphatase INPP5E which plays a role in the enrichment of PI4P in the ciliary membrane via dephosphorylation of PI(4,5)P_2_ leads to an accumulation of TULP3-dependent GPCR cargo in primary cilia (Badgandi et al., 2017).

INPP5E is also present in photoreceptors. Interestingly, INPP5E's localization in photoreceptors seems to be different from that documented for other ciliated cell types. Studies in primary cilia have shown INPP5E localization in the cilia, co-localizing with axonemal staining (Bielas et al., 2009; Jacoby et al., 2009). However, in photoreceptors INPP5E appears to be absent from the cilium, concentrated near the Golgi and proximal IS and does not appear to overlap with staining for centrin (Bielas et al., 2009; Hanke-Gogokhia et al., 2016). This is somewhat curious as INPP5E's localization in primary cilia is suggested to rely on the lipid binding chaperone PrBPδ which is also present in photoreceptors and is important for the OS localization of PDE6 and GRK1 (Zhang et al., 2007). It could be that another PI 5-phosphatase resides in the photoreceptor cilium in order to maintain low levels of PI(4,5)P_2_ and higher levels of PI(4)P. While PI(4)P is seen in both the IS and OS of photoreceptors, PI(4,5)P_2_ appears to be mostly excluded from the OS (Finkelstein et al., 2020), suggesting photoreceptors possess a mechanism for either enzymatically removing or excluding PI(4)P_2_ from the OS, similar to primary cilia. The PI 5-phosphatase ORCL (Oculocerebrorenal syndrome) is mutated in Lowe and Dent syndromes and was shown to localize to the OS of zebrafish photoreceptors (Luo et al., 2012). Perhaps photoreceptors have developed a system where ORCL and possibly other phosphatases work to remove PI(4,5)P_2_ from ciliary membrane and OS.

## Targeting of Outer Segment Plasma Membrane Proteins

Unlike the bulk of the phototransduction machinery that are mostly localized to the discs, two key proteins, the CNG channel and the Na^+^/Ca^2+^, K^+^ exchanger, which are found in a complex (Molday and Molday, 1998), are localized to the OS plasma membrane (Figure 5), raising the question of whether they use OS transport mechanisms that are distinct from the disc membrane destined proteins. The CNG channel is a heterotetramer consisting of three α1 and one β1 subunits (Weitz et al., 2002; Zheng et al., 2002; Zhong et al., 2002). The β1 subunit contains a glutamic acid rich GARP domain that binds to the tetraspanin protein, peripherin 2 (Poetsch et al., 2001), which itself is localized to the disc rims, thus providing structural stabilization between discs and the plasma membranes and confining the position of the CNG channels along the OS plasma membrane. The OS localization of the CNGβ1 subunit appears to rely on its glutamic acid rich GARP domain (Nemet et al., 2014). CNG interaction with peripherin 2 occurs within the IS (Ritter et al., 2011) and CNGβ1 is not located in vesicles found in the subretinal space of mouse rods lacking peripherin 2 (Spencer et al., 2019), leading to the speculation that trafficking of the CNG channel complex relies on association with peripherin 2. CNG channels are mislocalized to the IS in mouse rods lacking the endocytic adapter proteins Numb and Numb-like, which may redirect CNGα1 to endosomes (Ramamurthy et al., 2014).

However, a recent study claims to cast doubt on the role of peripherin 2 for CNG channel trafficking to the OS (Pearring et al., 2020). This study showed that CNG channels are delivered via the conventional secretory pathway, whereas peripherin 2 is delivered exclusively through the unconventional pathway (Tian et al., 2014). They show that the GARP domain possesses separate OS targeting sequence and peripherin 2 interaction domains. However, it cannot be ruled out that the Numb-mediated direction of the CNG channel to the late endosome, where transport pathways of CNG channels and peripherin 2 are thought to cross (Ramamurthy et al., 2014; Otsu et al., 2019), was disrupted by generating probes that only possessed specific domains.

In addition to trafficking to the OS, rod photoreceptor proteins must undergo an additional sorting between the disc membranes and the plasma membrane. A major unanswered question is how this separation occurs. It has been speculated that disc-plasma membrane protein sorting of CNG channels and the Na^+^/Ca^2+^, K^+^ transporter occurs when discs are enclosed within the plasma membrane (Spencer et al., 2020), presumably by a peripherin 2-Rom1-dependent mechanism (Conley et al., 2019b). However, more work is needed to support this idea.

Finally, disc and plasma membranes have distinct phospholipid content that may contribute to protein sorting between these membrane domains (Fliesler and Anderson, 1983; Albert and Boesze-Battaglia, 2005). Disc membranes contain equal amounts of PE and PC at 42 and 45%, respectively and 14% PS. the plasma membrane contains 10% PE, 65% PC, and 24% PS. Fatty acyl side chains are also highly divergent, with a ratio of 22:6 DHA in the disc vs. plasma membranes. The plasma membrane is enriched in cholesterol at 30 mole% relative to newly made discs which contain 0.3 mol% (Albert and Boesze-Battaglia, 2005). How phospholipid content is sorted between disc and plasma membrane is not understood.

## Peripheral Membrane Protein Compartmentalization Within the ROD Outer Segment

Peripheral membrane proteins reversibly interact with membranes, establishing an equilibrium between soluble and membrane bound states and are constantly in flux between the two. Membrane affinity is set by a number of physical factors, including hydrophobic, electrostatic, protein-protein and other binding interactions, alone or in combination. The majority of phototransduction components, including transducin, PDE6, GRK1, recoverin, RGS9, and Gβ5L are peripheral membrane proteins that are mostly localized to the OS. Some of these proteins undergo light-dependent redistribution from the OS to the IS (Calvert et al., 2006). Owing to the relative impermanence of membrane association, compartmentalization requires mechanisms in addition to the secretory pathways already discussed.

### Lipid Binding Chaperone Proteins Drive Outer Segment Localization of Some Peripheral Membrane Proteins

There are multiple lipid binding proteins present in photoreceptors, the most well-studied of which are PrBPδ (Norton et al., 2005) and Unc119a/b (Liu et al., 2007; Zhang et al., 2011a). PrBPδ is a prenyl binding protein with similar structural features to other lipid binding proteins, including Unc119 and RhoGDI (Zhang et al., 2007). IHC shows that PrBPδ is distributed throughout the photoreceptor. Labeling was significantly more intense in the IS extending to the synapse (Zhang et al., 2007). PrBPδ is thought to drive solubilization of prenylated proteins by depleting the soluble fraction, rather than extracting them from the membrane (Qureshi et al., 2018). It is required for the proper OS localization of PDE6 and for the stability of GRK1 (Zhang et al., 2007). The α and β subunits of PDE6 are farnesylated and geranylgeranylated, respectively (Anant et al., 1992) and GRK1 is farnesylated (Inglese et al., 1992).

Prenylation is required for PrBPδ binding to peripheral membrane proteins (Zhang et al., 2004). Prenyl moieties, however, possess different numbers of prenyl repeats, farnesyl with three and geranylgeranyl with four. This raises the question of whether PrBPδ prefers binding to one over the other. Studies that have addressed this question have conflicting results. A direct binding study using recombinant proteins showed that PrBPδ affinity for farnesylated GRK1 was ~30-fold higher than for geranylgeranylated GRK7 (Zhang et al., 2004). In contrast, mutation of the c-terminal leucine in the RPGR (retinitis pigmentosa GTPase Regulator) CAAX motif to methionine, which changes the c-terminal prenyl from a geranylgeranyl to a farnesyl, resulted in reduced ciliary localization, suggesting that PrBPδ has a lower affinity for the farnesyl moiety (Rao et al., 2016). A significant caveat to these studies, however, is that the extent of lipidation of these proteins was not clear. Moreover, carboxymethylation of prenylated proteins is another important modification (Inglese et al., 1992) that may influence the affinity of PrBPδ for prenylated proteins or the prenylated proteins for membranes and it is not clear what the methylation state was in these studies. Finally, variation in c-terminal sequences of the proteins may modulate affinity.

Unc119a is an acyl binding protein whose distribution in photoreceptors is primarily IS and synaptic with a small amount in the OS. It has been shown to bind both rod and cone Tα (Zhang et al., 2011a) and has been implicated in the light-dependent transport of Tα between OS and IS. Tα return to the OS upon transition from light to darkness is impaired in Unc119a knockout mice, but the transport from the OS to the IS upon transition from darkness to light is unaffected (Zhang et al., 2011a), suggesting that Unc119a is needed for transport to the OS but not for transport out. It is estimated that Unc119a is expressed in a molar ratio of 1:4 Unc119a:Transducin α (Sinha et al., 2013). Interestingly, Unc119a expression is reduced 2-fold in the GNAT1^−/−^ (Tα^−/−^) mouse (Sinha et al., 2013), suggesting there is a feedback on Unc119a expression based on acylated protein load. Efficient binding of Unc119a to Tα required N-terminal acylation as well as a peptide sequence, GAGASAEEKH, adjacent to the lipidation site (Zhang et al., 2011a). Unc119a, however, is not solely an acyl binding protein. It interacts with a number of non-lipidated proteins, including the SH2 and SH3 domains of some Src tyrosine kinases (Cen et al., 2003), Arl2 (Kobayashi et al., 2003), Arl3 (Veltel et al., 2008), CaBP4 (Haeseleer, 2008), and the synaptic protein RIBEYE (Alpadi et al., 2008).

A recent study showed that Unc119 expression levels in photoreceptors are influenced by its phosphorylation/dephosphorylation by casein kinase 2 (CK2) and the calcium regulated calcineurin (Chaya et al., 2019). The phosphorylation state of Unc119 affects its interaction with cullin 3-kelch-like 18 (Cul3-Klhl18) ubiquitin E3 ligase, which ubiquitinylates unphosphorylated Unc119, leading to its degradation. Unc119 levels were found to decrease in dark-adapted photoreceptors and knockout of Klhl18 resulted in reduced photoreceptor sensitivity, as determined with scotopic ERG, suggesting that the translocation of transducin α into the OS was reduced. Immunohistochemistry showed the Tα but not Tβγ levels were higher in the ISs of Klhl18^−/−^ mice. These results are suggested to potentially be of therapeutic relevance since FK506 and cyclosporine A, drugs that inhibit calcineurin, were protective of photoreceptor light damage.

AIPL1 is another putative lipid binding chaperone protein operating in rods (Sokolov et al., 2019). AIPL1 mutations are linked to LCA and affects both rods and cones (Sohocki et al., 2000). It has been shown to specifically bind farnesylated proteins in a yeast two hybrid study (Ramamurthy et al., 2003). AIPL1^−/−^ mouse expresses PDE6 subunits but they don't assemble properly and are likely degraded (Kolandaivelu et al., 2009). Recent results suggest that AIPL1 cooperates with PDE6γ subunit to catalyze proper PDE folding (Yadav et al., 2019). Thus, although apparently not directly involved in peripheral membrane transport, AIPL1 is key for PDE6 function in the rod OS.

Compartmentalization of peripheral membrane proteins that associate with lipid binding chaperones requires cargo displacement factors that release them, allowing association with destination membranes (Wright et al., 2011; Hanke-Gogokhia et al., 2016). The cargo displacement factors are the small GTPase ADP ribosylation factor-like proteins Arl2 and Arl3. Both Arl2 and Arl3 can displace cargo from PrBPδ, but only Arl3 can displace cargo from Unc119a (Ismail et al., 2012). Arl2 and Arl3 release cargo when in the GTP bound state, GTP binding to Arl3 is catalyzed by the guanine nucleotide exchange factor (GEF) Arl13b (Gotthardt et al., 2015; Zhang et al., 2016a). Upon release, the GTPase accelerating protein (GAP) Rp2 accelerates hydrolysis of the Arl3 bound GTP to GDP (Veltel et al., 2008; Evans et al., 2010). The GEF and GAP for Arl2 have not been identified.

Arl3 is found throughout the cell body and possibly in the CC, but is absent from the OS (Grayson et al., 2002; Hanke-Gogokhia et al., 2016; Wright et al., 2016). Rp2 is found throughout the cell body, where it is enriched on the basal bodies, perinuclear region, synapse and the periciliary membrane of photoreceptors (Evans et al., 2010; Holopainen et al., 2010), where membrane association is thought to be mediated by myristoylation (Evans et al., 2010). Arl3 KO in both retina and rod showed that Arl3 is important for ciliogenesis and ciliary maintenance as well as efficient localization of lipidated proteins such as PDE6, GRK1, Tα, and Tβγ to the OS (Hanke-Gogokhia et al., 2016).

In addition to releasing cargo from PrBPδ, Arl2 binds to BART/Arl2BP and is thought to recruit it to the basal bodies and periciliary membranes where it appears to be important for ciliogenesis and cilium maintenance (Davidson et al., 2013; Moye et al., 2018). Arl2 also binds tubulin specific chaperone cofactor D and is, thus, thought to regulate heterodimerization of α-, and β-tubulin (Bhamidipati et al., 2000). Arl2 is thought to be important for MT formation by regulating the soluble pool of tubulin, and thus is important for ciliary stability and control of axoneme length (Wright et al., 2018).

Arl13b is localized to the OS with enrichment in the proximal region (Hanke-Gogokhia et al., 2017; Dilan et al., 2019) and membrane association is mediated by double N-terminal palmitoylation (Roy et al., 2017). Retina specific mutations or deletions of Arl13b, which cause Joubert syndrome (Cantagrel et al., 2008), have a more severe phenotype than those impacting Arl3 (Hanke-Gogokhia et al., 2016). Retina specific Arl13b^−/−^ mice fail to form photoreceptor OSs and have improperly localized basal bodies (Dilan et al., 2019). Depletion of Arl13b in adult mouse rods causes accumulation of IFT88 at the proximal end of the cilia as well as mislocalization of Rhodopsin. Interestingly, PDE6, GRK1, and transducin localization are not affected, suggesting they transport to the ciliary photoreceptor OS in an Arl13b-independent way.

### Electrostatic Interactions and Peripheral Membrane Protein Compartmentalization

**Figure 7:**
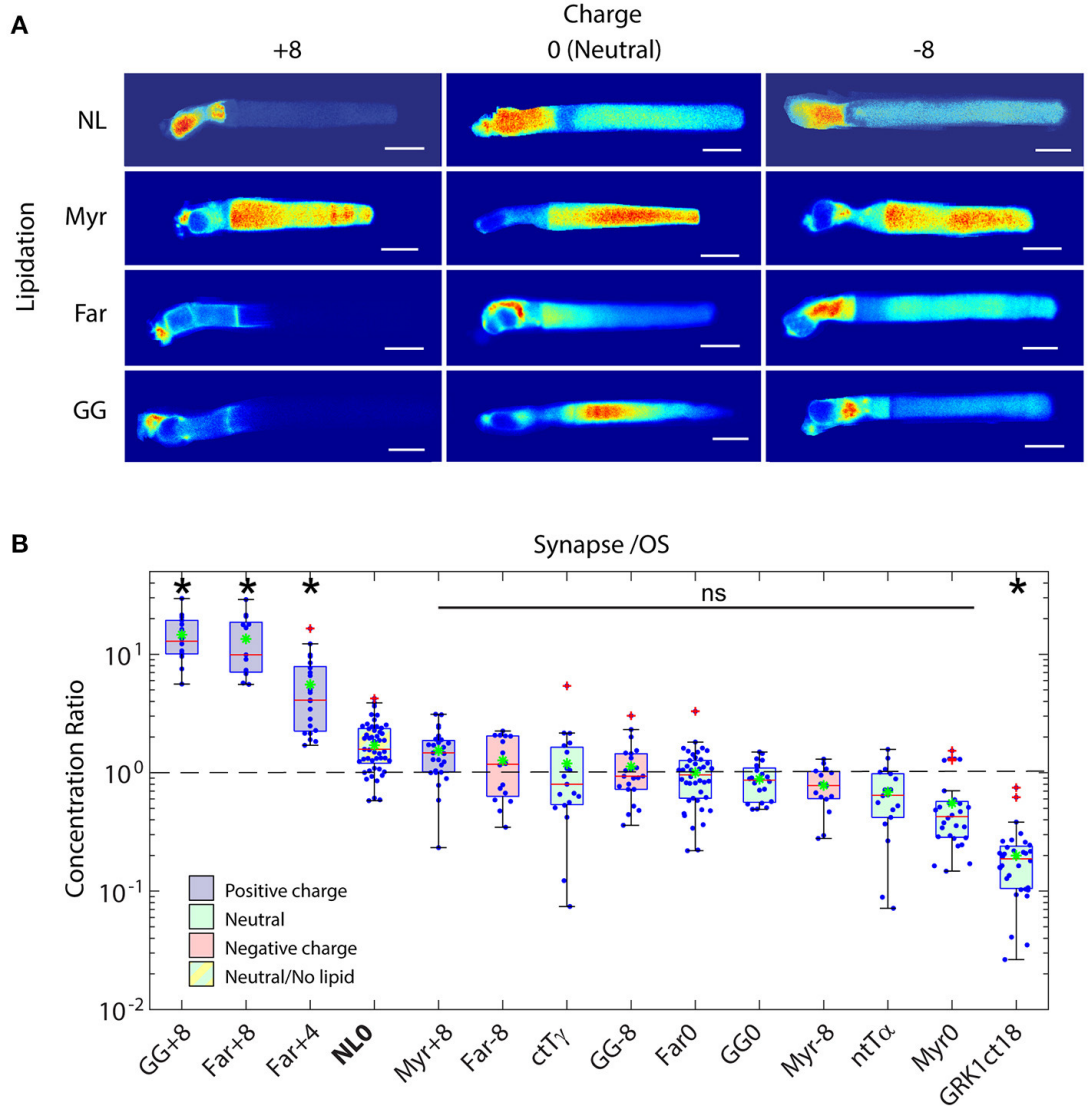
Role of electrostatic interactions in the compartmentalization of peripheral membrane proteins. **(A)** Montage of confocal images of living *Xenopus* rods expressing EGFP probes with indicated surface charge and lipidation motif. Note that none of the probes possess binding motifs for the lipid binding chaperone proteins, PrBPδ and Unc119. Significant OS localization of most probes shows that lipid binding chaperone proteins are not required for OS access and enrichment of peripheral membrane proteins. **(B)** Box-whisker plots of average fluorescence in the pre-synapse divided by average fluorescence in the OS shows that positively charged probes with prenyl lipids are depleted from the OS and enriched in the pre-synapse, while probes containing myristoylation and neutral or negative charge equally distributed between compartments. A probe consisting of EGFP fused to the myristoylation motif containing N-terminal 16 amino acids of Tα, which binds to Unc119, was not significantly more OS enriched than the Myr0 probe, which does not bind Unc119, suggesting that Unc119 association alone is not sufficient for OS enrichment. The probe containing the farnesylated C-terminus of GRK1, which does bind to PrBPδ, is more strongly OS localized, thus, PrBPδ tilted the equilibrium toward OS enrichment. However, presence of the Far0 probe in the OS shows that PrBPδ is not required for OS entry. Modified from Maza et al. (2019).

The model whereby lipid-binding chaperone proteins mediate transport of peripheral membrane proteins to the ciliary compartment implicitly or explicitly assumes that association with the lipid binding chaperones is required to enter the ciliary compartment and that tight membrane binding underlies the retention in the target compartment. Recently we showed in *Xenopus* rods that PrBPδ and Unc119 are not required for OS enrichment of all prenylated or acylated proteins (Maza et al., 2019). Moreover, using fluorescence recovery after photoconversion (FRAP) we showed that strong affinity of peripheral membrane proteins to target membranes is not required for compartmental enrichment. These results show that the basic distribution of peripheral membrane proteins in photoreceptors may be governed by diffusion and local binding and that the known lipid binding chaperones are not required for entry into the cilium (Figure 7). Although other, yet to be identified chaperone proteins cannot be ruled out, mass spectrometry analysis of pulldowns with lipidated-GFP probes did not identify obvious candidates (Maza et al., 2019). Compartment specific variation in membrane surface charge, protein content, ions as well as the lipid moiety and surface charge of the protein itself, therefore, may lead to differential compartmentalization of PMPs in photoreceptors, and presumably in primary cilia in general.

In (Maza et al., 2019) we also showed that a probe that consisted of EGFP and the c-terminal 18 amino acids of GRK1, which binds PrBPδ when farnesylated, are sufficient to drive effectively exclusive OS localization, despite the probe having relatively low membrane affinity. A probe with a neutral amino acid peptide linker bearing C-terminal farnesylation motif but that did not bind PrBPδ did not localize to the OS to nearly the same extent (Figure 7B, compare Far0 to GRK1ct18). This shows that the role of the lipid binding chaperone proteins is to tilt the equilibrium distribution of peripheral membrane proteins toward a specific compartment, rather than to deliver them to membranes where they bind with high affinity. There is a significant advantage to this mechanism. It allows peripheral membrane proteins within the OS, like GRK1, to equilibrate along the length of the OS, leading to equal numbers on each disc and, thus, uniform light responses throughout the OS. Indeed, amplitudes of dim light flashes are approximately uniform along the length of amphibian rod OSs (Baylor et al., 1979). Moreover, the localization to the OS is achieved without the non-specific blockade of the CC that would result from a soluble protein diffusion barrier.

### Lipid Switch Proteins

The distribution of some lipidated proteins in photoreceptors appears to rely on interactions that sequester lipid moieties into hydrophobic folds within the proteins themselves. Tβγ is a constitutive dimer with Tγ bearing a farnesyl moiety (Fukada et al., 1990; Lai et al., 1990). Farnesylation of Tγ is required for OS localization of the Tβγ dimer (Brooks et al., 2018). While PrBPδ has been implicated in proper Tβγ transport to the OS (Zhang et al., 2007), there is no published evidence for its involvement in light-dependent transport. Light-dependent transport of Tβγ appears to be disrupted by knockout of phosducin (Sokolov et al., 2004), a phosphoprotein in rods that undergoes light-dependent dephosphorylation and associates with Tβγ (Lee et al., 1987). Phosducin is phosphorylated by casein kinase 2 (CK2) in darkness (Humrich et al., 2003) and is dephosphorylated by protein phosphatase 2A in light (Brown et al., 2002). Phosducin appears to modulate the solubility of Tβγ through an interaction dependent sequestration of the Tγ farnesyl moiety between the β propeller blades 6 and 7 of Tβ (Gaudet et al., 1996; Loew et al., 1998), rather than direct binding to the lipid. Thus, phosducin operates a lipid switch on Tβγ. Despite the clear evidence that phosducin is involved in light dependent transport of Tβγ, there is no evidence that this transport impacts photoresponse kinetics or photoreceptor sensitivity (Sokolov et al., 2004; Krispel et al., 2007).

The calcium binding protein, recoverin, modulates GRK1 activity in a calcium and, thus, light dependent manner (Kawamura, 1993; Kawamura et al., 1993; Calvert et al., 1995; Chen et al., 1995; Klenchin et al., 1995). Knockout of recoverin in mouse rods shows that regulation of GRK1 leads to a moderate feedback control of light dependent PDE6 activity (Makino et al., 2004). Recoverin is N-terminally acylated (myristoylated) (Dizhoor et al., 1992). The acyl moiety is folded into a hydrophobic cleft on recoverin when calcium free (Tanaka et al., 1995), and extends into the bulk aqueous phase when Ca^2+^-bound (Ames et al., 1995b), through a mechanism known as the calcium-myristoyl switch (Zozulya and Stryer, 1992). The myristoyl moiety induces cooperativity in calcium regulation of GRK1 (Ames et al., 1995a; Calvert et al., 1995). Recoverin undergoes a modest light-dependent redistribution in mouse rods (Strissel et al., 2005), however Unc119a does not bind to recoverin (Zhang et al., 2011a), suggesting that the light-dependent redistribution is likely the result of changes in calcium concentrations and diffusion of recoverin in the calcium free state. Thus, the distribution of some peripheral membrane proteins relies on self-sequestration of lipid moieties as a result of specific binding interactions, and diffusion.

## Soluble Protein Compartmentalization in Photoreceptors

The most abundant soluble protein in rod photoreceptors, arrestin-1 (Arr1), is nearly equimolar to rhodopsin (Strissel et al., 2006; Song et al., 2011). The distribution of Arr1 in rods changes in response to light (Broekhuyse et al., 1985; Whelan and McGinnis, 1988; Peterson et al., 2003, 2005; Nair et al., 2005; Strissel et al., 2006). In dark-adapted photoreceptors, more than 90% of Arr1 is found in the IS. When exposed to bright light, 80% of Arr1 is found in the OS, where it binds to light-activated, phosphorylated rhodopsin, preventing further activation of the phototransduction cascade. The prevailing thought is that light-activated, phosphorylated rhodopsin serves as a binding sink to draw Arr1 into the OS. The role of phosphorylation of light activated rhodopsin, however, was brought into question when it was observed by the Chen group that Arr1 translocation in response to light was partially maintained in GRK1 knockout mice and mice where rhodopsin phosphorylation sites were mutated, which led to the speculation that Arr1 transport to the OS upon light exposure was mediated by an active, motor-driven mechanism (Mendez et al., 2003; Zhang et al., 2003). However, the sheer mass of Arr1 molecules moving to the OS in response to light, ~2 billion molecules in amphibian and ~75 million in mammalian rods, with a halftime of ~9–18 min (Peterson et al., 2003; Strissel et al., 2006), makes motor-driven transport to the OS unlikely (Calvert et al., 2006). The experiments by the Chen group were carried out under conditions that likely activated the entire pool of rhodopsin in the rods and, although the affinity of arrestin for light-activated unphosphorylated rhodopsin is ~35% of that for light-activated phosphorylated rhodopsin, it is ~8-fold higher than for dark-adapted rhodopsin (Nair et al., 2005). The study by Nair et al., that examined Arr1 localization to the OS at more moderate light intensities, showed that Arr1 transport to the OS, although present, was reduced in the GRK1^−/−^ and rhodopsin phosphorylation mutant mice (Nair et al., 2005). Moreover, Nair et al. showed that light dependent Arr1 transport was resistant to ATP depletion, suggesting that it is energy independent (Nair et al., 2005). Finally, employing our diffusion/active-transport/binding model (Maza et al., 2019), and considering the measured diffusivities of soluble proteins in the various rod compartments (Calvert et al., 2006, 2010; Najafi et al., 2012), we have predicted that the distribution of Arr1 in rods where the OS binding sinks had affinities corresponding to non-phosphorylated light-activated rhodopsin were nearly indistinguishable from that of rods with OS binding sinks with affinities corresponding to phosphorylated light-activated rhodopsin.

The notion that light-dependent Arr1 transport to the OS is independent of energy remains controversial, however. A more recent study in *Xenopus* and mouse showed that Arr1 transport to the OS in light-adapted rods was absent upon ATP depletion with potassium cyanide (KCN), and was restored with KCN washout and ATP supplementation (Orisme et al., 2010). The study further suggested that light-stimulated Arr1 transport is triggered by an ATP-dependent, PLC activated mechanism, perhaps mediated by D2 dopamine receptors. It is proposed that this pathway releases Arr1 from the IS by an unspecified mechanism and allows it to diffuse to the OS, where it is then retained by binding to light-activated rhodopsin. Together, these studies strongly support the diffusion to binding sink model for Arr1 transport to the OS of light-adapted rods, where the initial transport may require some form of release from the IS compartment. Interestingly, Strissel et al. observed that light stimulated Arr1 transport occurs at a threshold that activated ~3% of rhodopsins (Strissel et al., 2006). At light levels near this threshold the amount of Arr1 that transported to the OS was ten-fold higher than the number of rhodopsins activated by light, which is referred to as super-stoichiometric Arr1 transport, through an unidentified mechanism that requires phototransduction. It would be interesting to explore if the PLC-dependent mechanism might underlie this super-stoichiometric movement.

Several theories have been posited to explain the disproportionately IS biased distribution of Arr1 in dark-adapted rods, including a diffusion barrier at the base of the CC and tethering to IS structures by multiple binding partners, including NSF, enolase-1, and tubulin (Nair et al., 2004; Huang et al., 2010; Smith et al., 2011). However, studies of the dynamics of the soluble proteins EGFP and PAGFP have shown that there is no significant barrier impeding diffusion of soluble proteins up to ~80 kDa through the CC (Calvert et al., 2010; Najafi et al., 2012), and none of the Arr1 IS binding partners have sufficient concentration and localization to account for Arr1's distribution in the dark. This analysis led us to explore other mechanisms. We reasoned that the distribution of soluble proteins within cells will be governed by the available aqueous cytoplasmic spaces and interactions with binding partners (Peet et al., 2004). At first glance this appears to be a simple idea, however, the cytoplasm is crowded, and the available aqueous spaces are distributed among a heterogeneous patchwork of cytoplasmic structures. Highly organized organelles, like the lamellae of the ER and Golgi membranes, are interspersed with more open spaces. The meshwork of cytoskeletal components introduces sieves of variable mesh size. Together these structural features of the cytoplasm lead to partitioning of solutes into different cytoplasmic domains of cultured cells based on size (Luby-Phelps et al., 1986; Minton, 1992, 1997; Zimmerman and Minton, 1993; Janson et al., 1996; Zhou et al., 2008). In theory, partitioning of soluble molecules based on accessible cytoplasmic spaces may be a means of regulating cellular activities. However, quantifying that potential impact is difficult for most cells due to the spatial resolution limitations of light microscopes.

**Figure 8:**
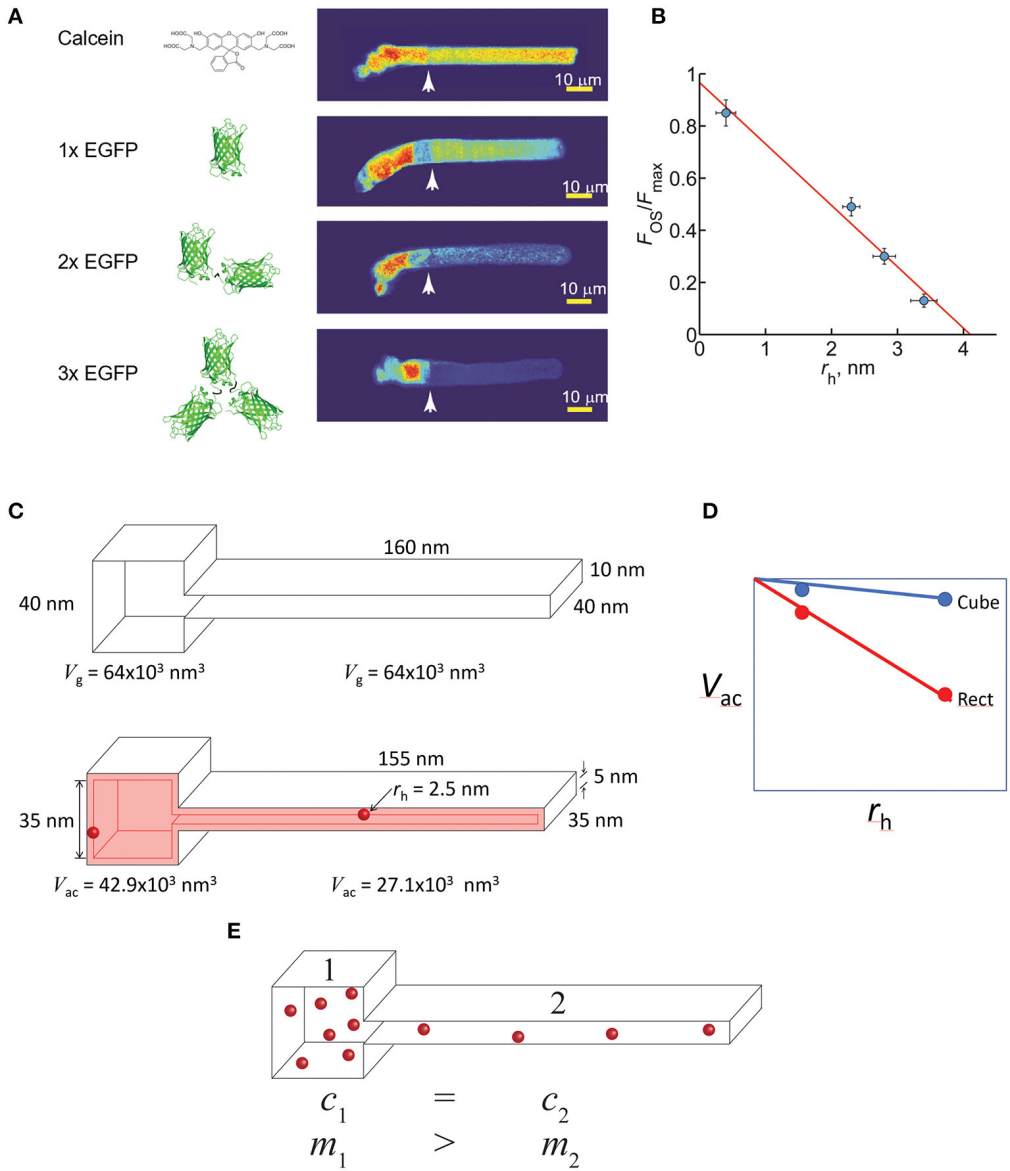
Steric volume exclusion and the compartmentalization of soluble proteins in photoreceptors. **(A)** The distribution of soluble molecules in *Xenopus* rods depends on the size of the molecule. Note that the conformation of the EGFP dimers and tetramer shown are only one of many possible. **(B)** The relationship of the ratio of the OS fluorescence to the maximum IS fluorescence scaled inversely and linearly with the estimated average hydrated radius of the molecules. This phenomenon can be explained by the asymmetrical reduction in the available aqueous volume of the differently shaped compartments caused by steric volume exclusion (i.e., loss of volume available to the center of mass of the molecule). **(C)** For example, two interconnected boxes have the same geometric volume (*V*_g_), but vastly different shapes. Introducing a spherical molecule reduces the geometry of both compartments, and thus the volumes accessible (*V*_ac_) to their centers of mass of the molecule. This reduction is larger for the rectangular compartment. **(D)** As the hydrated radius (*r*_h_) of the molecules increase, the reduction in *V*_ac_ falls more steeply for the rectangular compartment. **(E)** Since soluble molecules will equilibrate to equalize their concentrations (*c*) everywhere, the shape asymmetry will cause partitioning of the soluble molecules into the cubical compartment, where the total mass (*m*) will be higher. **(A,B)** modified from Najafi et al. (2012).

Amphibian photoreceptors provided a model cell where the impact of soluble protein partitioning by heterogeneous cytoplasmic spaces could be quantified (Figure 8) (Najafi et al., 2012). The large size of amphibian rods and the unique structure of photoreceptor OSs, with highly uniform spacing and close juxtaposition of the discs (~12 nm inter-disc spacing), allows quantification of the distributions of soluble molecules between the OS and the much less structurally constrained IS/cell body (Figure 8A). The ratio of molecules in the OS to that in the IS falls steeply, from ~0.85–~0.15, as the size of molecules increase from 600 Da to ~81 kDa (Figure 8B). Analysis of the size dependent distribution of molecules in the *Xenopus* rods agrees well with a model whereby the accessible volume within the OS declines much more steeply than the accessible volume in the IS due to steric interactions limiting the approach of the molecules to membranes and other surfaces (Figure 8C).

The steric volume exclusion effect operates in all cell compartments. Differences in the geometry of the compartments and the size of soluble proteins underlie the differential partitioning effect. To illustrate this, consider two interconnected cuboid compartments with identical geometrical volumes (Figure 8C). The volume accessible to the center of mass of a spherical protein is lower in the rectangular compartment than in the cubical. Moreover, the relationship between the accessible volume and the size of molecules falls more steeply in the rectangular compartment (Figure 8D). This difference in accessible volume means that the *effective concentration* of a *given number of molecules* in these two compartments will be higher in the rectangular compartment. This difference in effective concentration drives partitioning of the molecules into the cube shaped compartment. Thus, although the effective concentration of the soluble molecules will be the same in both compartments, the number of molecules will be lower in the rectangular compartment. In many signaling scenarios, it is the number of molecules that is most important. For instance, in photoreceptors the number of soluble Arr1 molecules in the inter-discal spaces dictate how quickly a light activated rhodopsin molecule is quenched.

The distribution by accessible volumes model predicts that Arr1 will be fivefold enriched in the IS relative to the OS (Najafi et al., 2012), somewhat less than the ~ 13-fold enrichment observed in dark-adapted rods. However, *in vitro* studies have shown that Arr1 can form dimers and tetramers (Granzin et al., 1998; Hirsch et al., 1999; Schubert et al., 1999; Shilton et al., 2002; Imamoto et al., 2003; Hanson et al., 2007, 2008; Kim et al., 2011), and the millimolar concentration of Arr1 in rods predicts the majority of Arr1 is in the dimer (~96 kDa) or tetramer (~192 kDa) forms. If the physiological form of Arr1 in dark-adapted rods is indeed dimer or tetramer, the dark-adapted partitioning of Arr1 can be explained simply by partitioning into the IS resulting from the heterogeneity of OS and IS structures and the steric volume exclusion effect. The distribution by steric volume exclusion-dependent partitioning between the OS and the rest of the cell is appealing for a number of reasons. First, it explains why Arr1 is found uniformly filling the entire non-OS compartments of dark-adapted rods, from the myoid region to the presynaptic spherule. Second, it overcomes the problem that the proposed non-rhodopsin Arr1 binding partners are not expressed to levels even nearly approaching that of Arr1. Third, although it has been proposed that Arr1 binds to microtubules (Nair et al., 2005; Orisme et al., 2010) and the BBSome protein BBS5 (Orisme et al., 2010), the sheer mass of Arr1 binding would appear to substantially inhibit other vital microtubule and BBS5 functions, including delivery of tubulin and other cargoes by IFT. Finally, distribution by steric volume exclusion offers the possibility of straightforward regulation of Arr1 distribution either through regulation of self-association or regulating the disc spacing in the OSs, which appear to elongate in response to light in the intact eye (Zhang et al., 2017).

## Concluding Remarks

Photoreceptor OSs are modified primary cilia with highly elaborated membrane systems that serve to efficiently capture photons and transduce them into electrical signals. To support this role, proteins involved in phototransduction and maintenance of the OS structure must be transported to the OS, an especially daunting problem for photoreceptors because of the sheer mass of protein and membranes that are required for daily renewal of 10% of the OS. Over the last decade and a half, significant advances in our understanding of the molecular players involved in photoreceptor OS genesis and protein transport have been made due to a refreshed examination of this problem using focused mouse genetic models, advanced proteomics and the development of sophisticated approaches to high resolution imaging of the CC/photoreceptor transition zone, including cryo-EM tomography and super-resolution fluorescence microscopy. Recent advances in live cell imaging in photoreceptors, primary cilia and flagella, however, have begun to challenge previous notions of the mechanisms underlying ciliary protein transport, ciliary construction and maintenance in health and disease. We are just at the beginning—further development of high spatial and temporal resolution approaches to quantifying the dynamics of protein and membrane transport in living photoreceptors and other cilia represents a critical area for advancing the field.

## Author Contributions

CB and HM: Researched, wrote draft, and edited manuscript. PC: Proposed review article, researched, wrote draft, edited manuscript, and generated figures. All authors contributed to the article and approved the submitted version.

## Conflict of Interest

The authors declare that the research was conducted in the absence of any commercial or financial relationships that could be construed as a potential conflict of interest.

## Abbreviations

AIPL1: Aryl hydrocarbon receptor interacting protein like 1
ARL13B: ADP-ribosylation factor-like protein 13B
Arl2: ADP-ribosylation factor-like protein 2
Arl3: ADP-ribosylation factor-like protein 3
Arr1: Arrestin 1
ATP: Adenosine triphosphate
BBS: Bardet-Biedl syndrome
CC: connecting cilium
COS: Cone outer segment
CNG1: Cyclic nucleotide-gated channel 1
DHA: Docosahexaenoic acid
PAGFP: Photoactivable green fluorescent protein
EGFP: Enhanced green fluorescent protein
ER: Endoplasmic reticulum
GARP: Glutamic acid rich protein
GC1: Guanylate cyclase-1
GDP: Guanosine diphosphate
GPCRs: G-protein coupled receptors
GRK1: G Protein-coupled receptor kinase 1
GTP: Guanosine triphosphate
IC3: intracellular loop 3
IFT: Intraflagellar transport
INPP5E: Inositol polyphosphate-5-phosphatase E
IS: inner segment
Kif3A: kinesin family member 3A
OS: outer segment
PC: phosphatidylcholine
PDE6: rod photoreceptor phosphodiesterase 6
PE: Phosphatidylethanolamine
PI4P: Phosphatidylinositol 4-phosphate
PI(4,5)P2: Phosphatidylinositol (4,5)-bisphosphate
PLC: Phospholipase C
PrBPδ: Prenyl-binding protein delta
PRCD: Progressive Rod-Cone Degeneration
Rho: Rhodopsin
ROS: Rod outer segment
RIS: Rod inner segment
RTC: Rhodopsin transport carrier
Smo: Smoothened receptor
SSTR3: Somatostatin receptor 3
STX3: Syntaxin 3
T: G protein transducin
Tα: alpha subunit of transducin
Tβγ: heterodimer of transducin beta and gamma subunits
TEM: Transmission electron microscope
TGN: Trans-Golgi network
TZ: Transition zone
Unc119: Uncoordinated 119.
